# Halogen‐Bonded Liquid Crystal Elastomers as Initiator‐Free Photochemical Actuators

**DOI:** 10.1002/adma.202504551

**Published:** 2025-05-28

**Authors:** Hongshuang Guo, Roshan Nasare, Chen Liang, Kim Kuntze, Eugene M. Terentjev, Arri Priimagi

**Affiliations:** ^1^ Smart Photonic Materials Faculty of Engineering and Natural Sciences Tampere University P.O. Box 541 Tampere FI‐33101 Finland; ^2^ Department of Applied Physics Aalto University P.O. Box 15100 Espoo FI 02150 Finland; ^3^ Cavendish Laboratory University of Cambridge J. J. Thomson Avenue Cambridge CB3 0HE UK

**Keywords:** azobenzene, halogen bonding, liquid crystal elastomer, photoactuation, soft robotics

## Abstract

Photochemically driven liquid crystal elastomer (LCE) actuators require precise arrangement and sufficiently high concentration of photoswitchable molecules for effective actuation. Achieving both high photoswitch content and a high degree of molecular alignment has been challenging especially in thick samples, but is essential for future applications in soft robotics, biomedicine, and photonic technologies. In this work, this issue is addressed by combining dynamic halogen bonds with Aza–Michael addition‐based polymerization, creating azobenzene‐containing LCEs with multimodal actuation capabilities. These highly directional supramolecular interactions eliminate the need for a photo‐initiator in the LCE fabrication process, enabling control over the azobenzene content over a wide range while maintaining a high degree of molecular alignment and dynamic programming ability. The potential of this approach is demonstrated through proof‐of‐concept examples such as light‐guided rolling movement and underwater gripping, underscoring the versatility of the weak, dynamic halogen bonds in advancing the supramolecular design of multimodal soft actuators.

## Graphical abstract

The combination of dynamic disulfide bonds and halogen bonds in chain‐extended liquid crystal elastomers enables the fabrication of photochemical actuators without the need for a photoinitiator, advancing the design of multimodal soft actuators that can be reprogrammed and reprocessed through supramolecular functionalization.

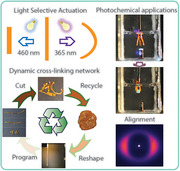

## 1 Introduction

Soft actuators are made of flexible and compliant materials, representing a significant shift from conventional rigid actuators.^[^ ^1^, ^2^ ^]^ In fields like small‐scale robotics and biomedicine, soft actuators enable applications that are beyond the capabilities of their rigid counterparts.^[^ ^3^ ^]^ At the core of these applications are stimuli‐responsive materials that serve as essential building blocks for soft actuators. Designed to respond to external stimuli such as light, heat, electric or magnetic fields, or chemical cues, stimuli‐responsive materials impart dynamic behavior to soft actuators, equipping them with characteristics that can be considered intelligent from a simplified perspective.^[^ ^4^, ^5^, ^6^, ^7^ ^]^ Among these, light‐responsive actuators are particularly appealing, as they offer wireless control over material motions with high spatiotemporal resolution, self‐sustained feedback‐driven actuation, and miniaturization of the robotic structures.^[^ ^8^, ^9^, ^10^, ^11^ ^]^ Combined with the versatility of polymer‐based materials such as hydrogels,^[^ ^12^, ^13^ ^]^ liquid crystal elastomers (LCEs),^[^ ^14^ ^]^ or bilayer carbon materials,^[^ ^15^, ^16^ ^]^ recent progress in light‐driven, responsive‐material‐based robotics has propelled the development of systems that are able to navigate, adapt, and autonomously make simple decisions in intricate environments.

Among light‐responsive actuators, LCEs stand out as a promising choice due to the relative ease of controlling the alignment of liquid crystal molecules during polymerization.^[^ ^17^, ^18^, ^19^, ^20^ ^]^ Upon light irradiation, the spontaneous molecular alignment is disrupted but would recover its programmed order on removing the light.^[^ ^21^, ^22^ ^]^ This creates anisotropic strains in the loosely crosslinked polymer network, which results in reversible macroscopic actuation of free‐standing LCE strips. These distinctive features have enabled mimicking advanced biological functions such as camouflage,^[^ ^23^ ^]^ self‐healing,^[^ ^24^ ^]^ collective behavior with emergent properties,^[^ ^25^ ^]^ and skin‐like functionalities.^[^ ^9^ ^]^ Light‐responsive LCEs can be actuated photothermally, photochemically, or through a combination of these.^[^ ^26^ ^]^ In the photothermal response, light absorption generates heat, which distorts the molecular alignment and leads to material deformation.^[^ ^27^ ^]^ Upon ceasing the irradiation, the deformed state quickly relaxes to the initial state.^[^ ^28^, ^29^, ^30^ ^]^ The photochemical response arises from the covalent attachment of molecular photoswitches such as azobenzenes into the polymer network.^[^ ^31^, ^32^, ^33^, ^34^, ^35^, ^36^ ^]^ The anisotropic molecular alignment amplifies the molecular‐level motion (photoisomerization) into the macroscopic scale, generating anisotropic stress and reversible actuation. The process is isothermal, making photochemical actuation efficient even in submerged environments.^[^ ^37^ ^]^ Another important benefit of photochemical actuators is the ability to retain the deformed state for extended periods, determined by the lifetime of the metastable state of the molecular switch.^[^ ^38^, ^39^ ^]^

While the photothermal effect can be easily achieved by adding small amounts of light‐absorbing dopants into the LCE, efficient photochemical actuation is more challenging. This is because for efficient photomechanical response, the molecular switches are oftentimes coupled to the polymer network, in a relatively high content to translate the molecular‐level motions into macroscopic actuation. However, increasing the photoswitch concentration can pose challenges for conventional photopolymerization‐based sample fabrication methods by reducing polymerization efficiency if the absorption spectra of the switch and the photoinitiator overlap.^[^ ^40^, ^41^ ^]^ This issue can be particularly severe for thick samples, and in extreme cases, the presence of photoswitches can scavenge the polymerization process altogether. It has been addressed using supramolecular functionalization strategies, leveraging spontaneous non‐covalent crosslinks in solidifying the network and hence eliminating the need for photo‐curing. This approach has been prominent in devising photochemical actuators that can be programmed into complex photoactuable shapes.^[^ ^42^, ^43^ ^]^ The existing strategies have relied on hydrogen bonding and reversible Diels–Alder reaction. Expanding the range of supramolecular interactions is essential for enhancing the performance of photochemically addressable LCEs. Different non‐covalent interactions may exhibit varying sensitivity to environmental factors such as water or chemical exposure, potentially uncovering new design principles and broadening the application potential of LCEs. With this in mind, we turn our attention to halogen bonding.

Halogen bonding (XB) is an attractive interaction between an electron‐deficient region of a polarizable halogen atom attached to an electron‐withdrawing moiety (the σ‐hole) and a nucleophile.^[^ ^44^, ^45^ ^]^ As a highly directional and tunable interaction, XB stands out from the more widely utilized hydrogen bonding, and it has been effectively utilized in the design of supramolecular co‐crystals, liquid crystals, and functional polymers.^[^ ^46^, ^47^, ^48^ ^]^ Importantly, XB is stable in aqueous environments, making it valuable, e.g., as supramolecular passivation layers in optoelectronic components,^[^ ^49^, ^50^ ^]^ and potentially in supramolecular constructs exhibiting efficient underwater robotic functionalities.^[^ ^51^ ^]^ We have recently introduced XB as a novel supramolecular interaction in photothermally driven LCEs.^[^ ^52^ ^]^ By employing diiodotetrafluorobenzene as a supramolecular crosslinker in chain‐extended Aza–Michael addition‐based LCEs, we demonstrated reversible actuation combined with arbitrary shape‐programming and self‐healing capabilities. This leads us to intriguing questions: Can we fabricate high‐azobenzenecontent, XB‐functionalized LCEs without relying on photopolymerization? If so, can we produce thick samples—up to hundreds of micrometers— with high‐azobenzene‐content while still achieving a high degree of molecular alignment?

In this work, we address these questions by incorporating azobenzenes into initiator‐free, halogen‐bond‐crosslinked polymer main chains to fabricate photochemically driven LCE actuators. The chemical design allows for flexible control over the azobenzene content, and highly ordered LCEs can be obtained even when using an equimolar ratio between azobenzene and the non‐absorbing reactive mesogen (RM82). The material design combines weak XB crosslinks and strong, yet dynamic, disulfide‐containing crosslinks, providing the mechanical strength and flexibility needed for mechanical programming, self‐healing, and reversible photochemical actuation. Proof‐of‐concept robotic demonstrations, such as shape‐programmable rolling in the air and underwater, as well as grippers in submersed conditions, are shown. We also leverage the properties of dynamic bonds to showcase the recyclability and reprocessing of the material through compression molding.

## 2 Results and Discussion

**Figure 1:**
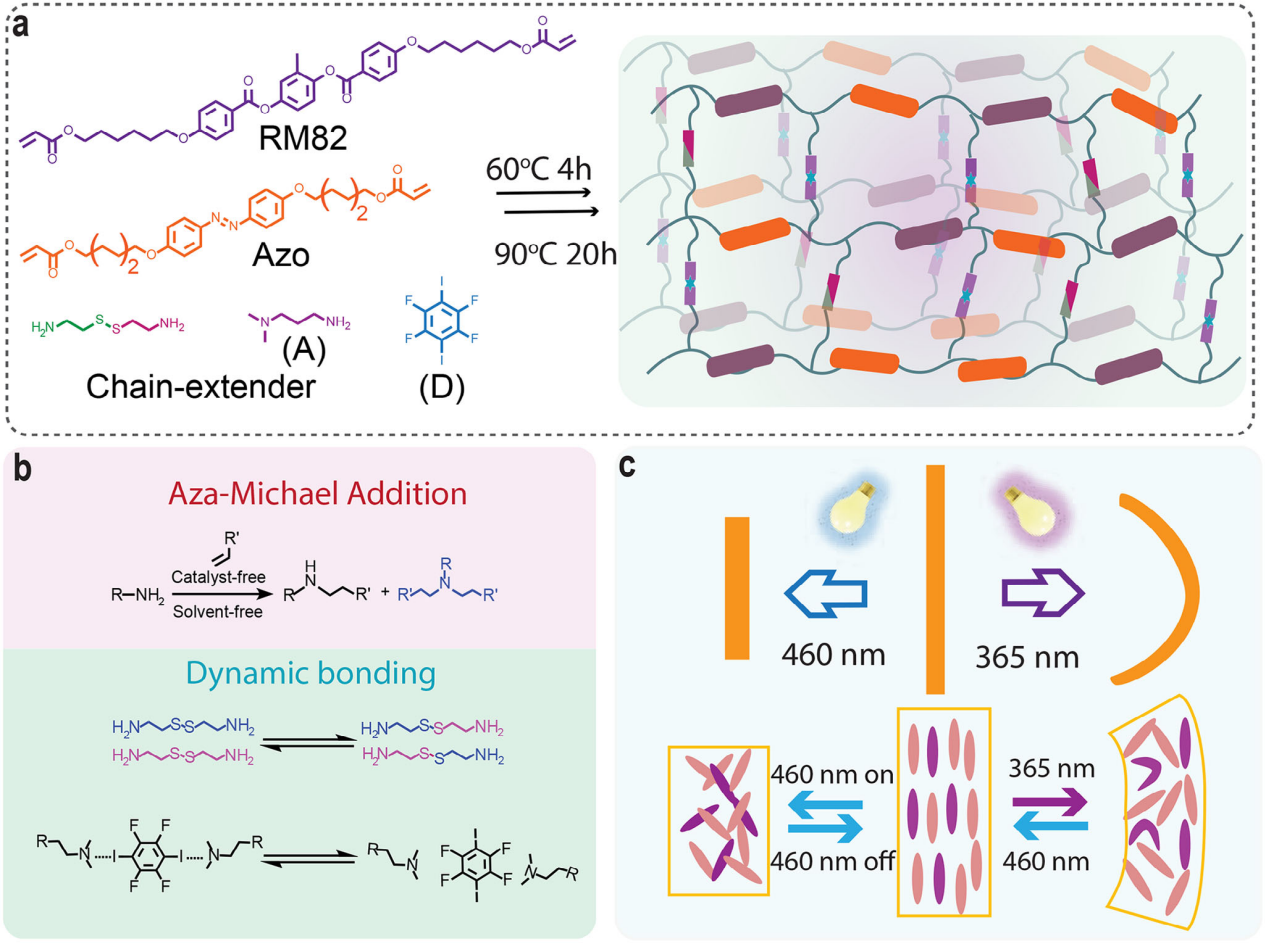
The Material Design Concept. a) The chemical composition of the LCE used and schematic representation of the corresponding LCE network. b) Schematic diagram of the Aza–Michael addition reaction and dynamic bonding mechanism. c) Schematic representation of the photothermal contraction and photochemical bending of LCEs under light exposure at different wavelengths.

Shortly after the initial reports on Aza–Michael addition‐based, chain‐extended LCEs,^[^ ^53^, ^54^ ^]^ it became evident that these materials offer new possibilities beyond surface‐alignment‐dictated shape morphing. They also facilitate modification through dynamic bonding,^[^ ^55^, ^56^, ^57^, ^58^ ^]^ enabling advanced features such as shape programming and self‐healing. We expand on this concept utilizing RM82 and 4,4′‐Bis(6‐acryloyloxyhexyloxy)azobenzene (Azo), as the polymerizable mesogens, and cystamine and N,N‐dimethylaminopropylamine as chain extenders (**Figure** **1**a). Cystamine serves as a dynamic covalent crosslinker,^[^ ^59^, ^60^ ^]^ enabling bond exchange at high temperatures, as will be shown with creep and stress relaxation experiments. N,N‐dimethylaminopropylamine acts as an XB acceptor (**A**),^[^ ^52^ ^]^ which is combined with 1,4‐diiodotetrafluorobenzene, a common bifunctional XB donor (**D**). Here, halogen bonds are used for post‐programming to obtain a high orientation parameter. This combination facilitates supramolecular crosslinking within the dynamic stimuli‐responsive LCE network (Figure 1a; Figure S1a, Supporting Information). Through this combination, we eliminate the need for photopolymerization (and photoinitiators) in the LCE fabrication process (Figure 1b).^[^ ^42^, ^43^, ^52^, ^55^ ^]^ This is particularly beneficial when devising photochemical actuators that require high azobenzene concentration.

The Aza–Michael addition reaction was performed using a mixture with proportions of X:Y:3:4:2 for Azo, RM82, cystamine, **A**, and **D**, respectively, ensuring that X + Y = 10. The resulting elastomer is labeled as LCE‐Azo X%, where X% indicates the proportion of Azo to Azo+RM82 (X * 100/X + Y) (Figure S1b, Supporting Information). The preparation of halogen‐bonded photochemical LCEs follows a two‐stage process (Figure S2, Supporting Information). First, the monomer mixture is filled into a PVA‐coated cell (thickness 550 µm) at 100 °C, cooled to 60 °C and kept at that temperature for 4 h to initiate chain extension. The sample is subsequently heated to 90 °C for 20 h to complete the polymerization, yielding a solid LCE film.^[^ ^55^ ^]^ The films are then cut into strips with desired dimensions, heated to 100 °C to break the halogen bonds, stretched to ca. 100%, and kept stretched for 3 days at room temperature. The resulting LCEs undergo photochemically induced bending/unbending in response to UV/visible light, while exposure to visible light leads to photothermal contraction of the uniaxially aligned LCE strip (Figure 1c).

**Figure 2:**
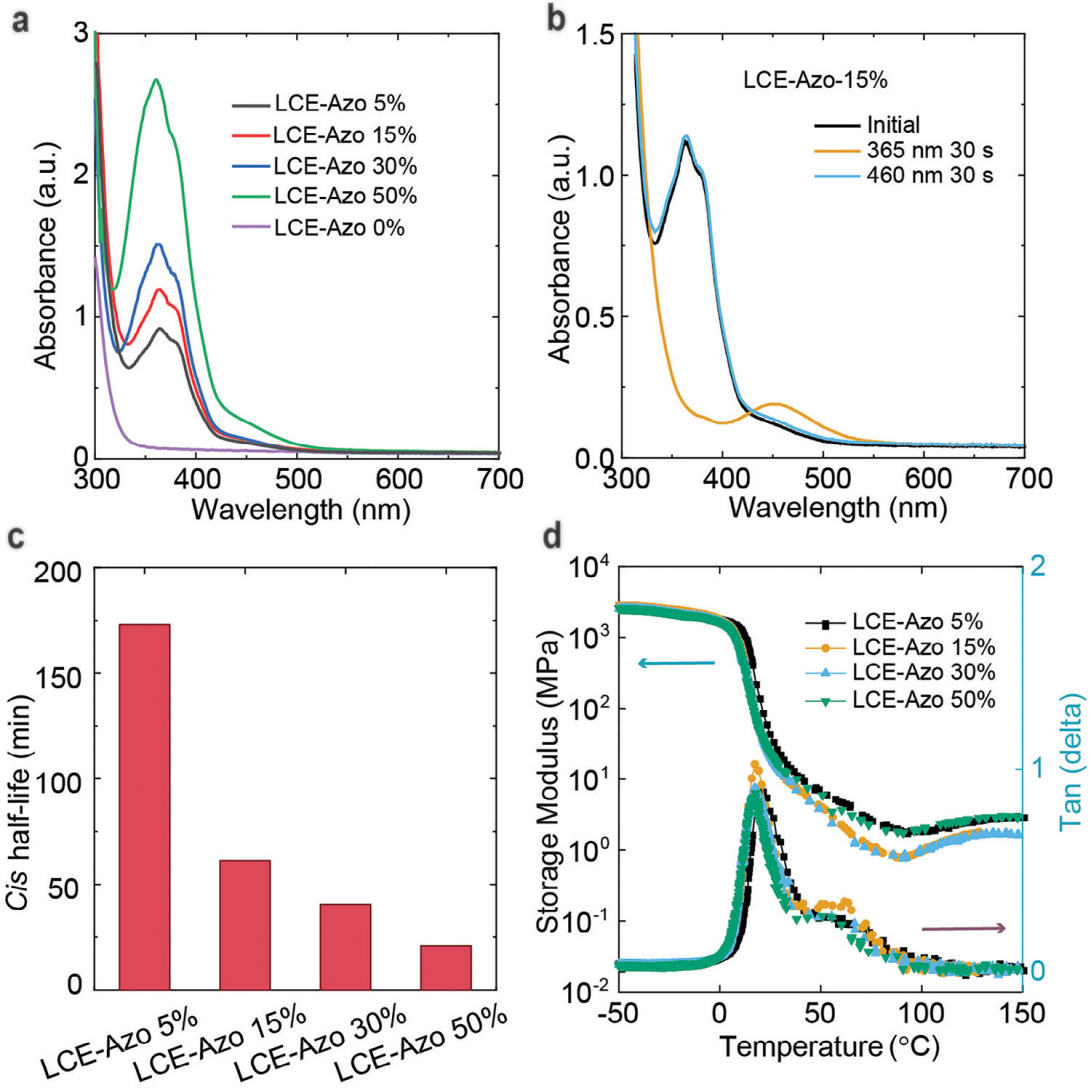
Material Characterization. a) UV–vis spectra of the LCE‐Azo X% from 300–700 nm, confirming that increasing azobenzene content in the polymerizable mixture translates into higher azobenzene content in the polymerized LCE. b) The π–π* and n–π* bands of Azo can be used to monitor the photoisomerization process, as shown for LCE‐Azo 15% under subsequent 365 nm (35 mW cm^−2^, 1 min) and 460 nm (45 mW cm^−2^, 1 min) irradiation. c) The *cis* half‐life of LCE‐Azo X% at room temperature depends drastically on Azo content. d) Storage modulus (*E′*) and loss tangent (*tan δ*) of LCE‐Azo X% as a function of temperature.

The initiator‐free fabrication method, enabled by the combination of dynamic covalent disulfide and supramolecular XB crosslinks, allows for easy control over the Azo content, up to equimolar ratio between Azo and RM82 (LCE‐Azo 50%). Attempts to increase the Azo content further and remove RM82 altogether were unsuccessful. The resulting samples (LCE‐Azo 100%) were opaque, crystalline, and lacked LC order after stretching (Figure S3, Supporting Information). The presence of XB between **A** and **D** was confirmed using Raman spectroscopy, evident from a red‐shift of the uncomplexed C─I bond (157 cm^−1^) by *ca*. 10 cm^−1^ (Figure S4a,b, Supporting Information).^[^ ^61^, ^62^, ^63^ ^]^ Increasing the Azo content in the LCE was confirmed through UV–vis spectroscopy by tracking the π–π* (365 nm) in unstretched LCE films (**Figure** **2**a), which progressively intensified up to the equimolar ratio between Azo and RM82. Azobenzene photoswitching in the LCE was confirmed by following the changes in the π–π* and n–π* band upon alternating UV and visible illumination (Figure 2b; Figure S5a–c, Supporting Information). The *cis* half‐life of LCE‐Azo X% is presented in Figure 2c and Figure S6a–d (Supporting Information), showing a systematic decrease with increasing Azo content. This trend is likely attributed to a combination of steric constraints, changes in the environmental polarity, and enhanced Azo–Azo intermolecular interactions.^[^ ^64^ ^]^ This can also be observed from the UV absorption, where the π–π* transition from 366 nm shifts to 360 nm (Figure 2a). This is further supported by the infrared spectra shown in Figure S7a–c (Supporting Information), where the N═N (1450–1550 cm^−1^) and C–N (1200–1300 cm^−1^) stretching bands display red shifts. These shifts become more pronounced with increasing Azo content, highlighting the role of Azo–Azo interactions.

We then examined the thermodynamic and mechanical properties of LCE‐Azo X%. The glass transition temperature (*T_g_*) decreases gradually with increasing Azo content, from 13 to 7 °C, as measured using differential scanning calorimetry (DSC; Figure S8a–d, Supporting Information; **Table** **1**). This trend can be rationalized through weakening of the interactions between the mesogenic RM82 molecules by gradually increasing the content of the non‐mesogenic Azo molecules. The mechanical properties were assessed through uniaxial stretching tests on pristine, unstretched samples (16 × 2 × 0.5 mm^3^). The elastic moduli, tensile strengths, and fracture strains were comparable, with no clear dependence on the Azo content (Table 1). Dynamic mechanical analysis (DMA) was performed to evaluate the viscoelastic behavior of LCE‐Azo X% (Figure 2d). The thermograms reveal that the inflection points of the storage modulus (*E*) and the peaks of the loss tangent (*tan δ*) occur at ≈20 °C for all compositions. This suggests that under dynamic testing conditions, no significant difference in *T_g_* can be observed. All samples display a nematic range between 20 and 90 °C, and all samples exhibit a flat rubbery plateau as the temperature increases.

**Table 1:** Mechanical properties and glass transition temperature (*T_g_*) of LCE‐Azo X%. The elastic moduli, tensile strengths, and fracture strains have been obtained by averaging over three different samples.

Code	E_Y_ [MPa]	σ_max_ [MPa]	ɛ_max_ [%]	*T_g_* [°C]
LCE‐Azo 5%	5.2 ± 0.3	7.2 ± 1.0	180 ± 15	13
LCE‐Azo 15%	4.4 ± 0.7	4.5 ± 0.7	177 ± 9	12.3
LCE‐Azo 30%	4.5 ± 0.8	4.1 ± 0.5	148 ± 4	9.1
LCE‐Azo 50%	4.0 ± 0.5	3.7 ± 0.5	166 ± 10	7

**Figure 3:**
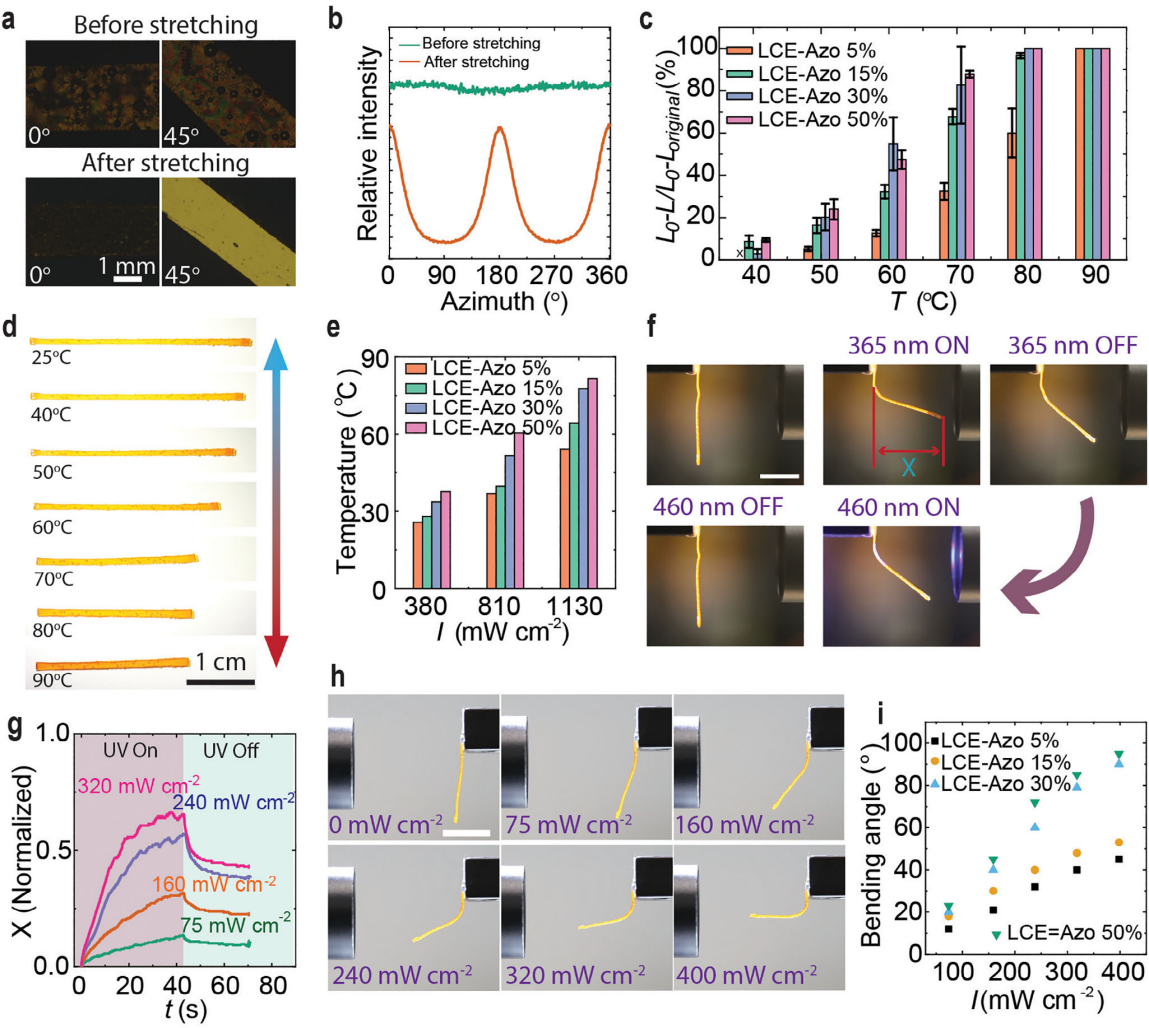
Thermal and photoactuation. a) Polarized optical micrographs of the LCE‐Azo 15% before and after stretching (100%). b) 1D azimuthal scan profiles for LCE‐Azo 15% strip before and after stretching (100%). c) Contraction under uniform heating. *L_0_* stands for the initial length after stretching, *L* is the contracted length, and *L_original_* is the initial length before stretching. d) Photographs of thermal actuation of the LCE‐Azo 15% when heated from 25 to 90 °C. e) Light‐induced temperature increase of LCE‐Azo X% under 460 nm irradiation. f) Photoinduced bending and unbending of LCE‐Azo 15% strip under 365 nm (240 mW cm^−2^, 40 s) and 460 nm (160 mW cm^−2^, 40 s) irradiation, respectively. X represents the distance the vertex moves in the X‐axis direction. Scale bar: 1 cm. g) UV‐light‐induced bending kinetics of LCE‐Azo 15% under different irradiation intensities. X was defined in (f). The length of the X‐directional movement of the tracking vertex is divided by the length of the strip. h) UV‐light‐induced bending of LCE‐Azo 30% at different light intensities. Scale bar: 1 cm. i) Bending angle of LCE‐Azo X% caused by photochemical deformation at different light intensities.

The LCE‐Azo X% are initially in an isotropic polydomain state after polymerization, as evidenced by the lack of contrast during sample rotation in polarized optical micrographs (**Figure** **3**a). Upon uniaxial stretching (100%), the film becomes strongly anisotropic due to the alignment of LC mesogens along the stretching direction (Figure 3a; Figure S9, Supporting Information). The anisotropic molecular orientation was verified using wide‐angle X‐ray scattering (WAXS). Before stretching, the WAXS pattern displayed a ring‐shaped isotropic scattering and a featureless curve, indicative of random molecular orientation (Figure 3b; Figure S10, Supporting Information). After stretching, the samples showed strong scattering peaks perpendicular to the stretching direction, confirming a high degree of monodomain molecular alignment during stretching.^[^ ^23^, ^65^ ^]^ The order parameter of the stretched LCE‐Azo X% was determined using the Hermans–Stein orientation distribution function,^[^ ^66^, ^67^ ^]^ with values ≈0.7 for all the samples. WAXS analysis at elevated temperatures revealed a gradual loss of molecular alignment with a distinct order‐to‐disorder phase transition. For LCE‐Azo 15%, the order parameter decreased to 0.54 at 50 °C, and further to 0.35 at 75 °C (Figure S11, Supporting Information).

We assessed the actuation performance of the stretched samples by measuring contraction and bending angles as a function of temperature and light intensity. In the absence of an external load, all samples contracted back to their original length when heated to 90 °C and returned to the elongated state after cooling (Figure 3c). The samples shrink within a few seconds and recover their original shape in the same time scale. Photographs in Figure 3d and Figure S12 (Supporting Information) illustrate the thermally induced contraction for LCE‐Azo 15% and LCE‐Azo 30%, respectively. Light‐induced actuation was first examined under 460 nm irradiation, a wavelength absorbed by the n–π* band but driving the samples to a *cis*‐rich state, causing photothermal actuation. As expected, the temperature increase depended on both the light intensity and the Azo content, exceeding 80 °C under proper experimental conditions (Figure 3e; Figure S13, Supporting Information).

The photochemical actuation of LCE‐Azo X% was investigated under 365 nm illumination, which causes *trans–cis* isomerization (Figure 2b). Due to light attenuation through the sample, a *cis* isomer gradient is formed through its thickness, leading to bending. This gradient induced a gradual change in molecular alignment across the sample thickness, generating internal stress and strain gradients that lead to macroscopic bending. When the irradiation is ceased, the bent state is only partially retained (Figure 3f; Video S1, Supporting Information). This indicates that, in addition to the *cis* gradient, the thermal gradient from photoheating also contributes to the bending, and as the material cools down after irradiation, it partially unbends. The actuation response is intensity‐dependent (Figure 3g–i; Figures S14–S16, Supporting Information), with higher Azo content leading to greater bending at fixed intensity. The bending angle is influenced by several factors, such as light intensity, exposure time, and the degree of alignment within the material. The azobenzene content affects both the formation of *cis*‐gradient and the thermal effect caused by light absorption, thus playing a multifaceted role in the photoinduced deformation. The material bends in a direction that minimizes internal strain, with the bending angle increasing with the extent of isomerization. In thick samples, bending under UV irradiation typically occurs in tens of seconds, and a similar timescale is required to return to the initial state under visible light. In dark, the relaxation (unbending) time is often governed by the *cis*‐lifetime of the azobenzene molecules. The photochemical bending is reproducible and shows only moderate fatigue over 60 bending/unbending cycles (Figure S17, Supporting Information). The photoheating effect becomes more pronounced with increasing Azo concentration. However, the bent state remains stable at room temperature for extended periods in all samples (Figure S18, Supporting Information). There is no clear correlation between the *cis* lifetime (Figure 2c; Figure S6, Supporting Information) and the stability of the bent state. Instead, the supramolecular crosslinks seem to “lock” the shape change, as has been previously observed in 4D‐printed photoactuators crosslinked via hydrogen bonding.^[^ ^42^ ^]^ The original unbent state can be restored through 460 nm illumination, which triggers both photothermal heating and *cis–trans* isomerization. Additionally, when subjected to an external load, LCE‐Azo 15% contracts under both 365 and 460 nm irradiation, demonstrating the capability to lift over a thousand times its own weight (Figure S19 and Video S2, Supporting Information). To further explore the influence of light on the material's mechanical properties, we investigated the effect of UV irradiation on Young's modulus (Figure S20, Supporting Information). Exposure to UV light led to a decrease in the modulus of LCE‐Azo 15%, while the modulus of the control sample without azobenzene, LCE‐Azo 0%, remained nearly unchanged under the same conditions. This suggests that the modulus reduction is driven by the *trans*–*cis* isomerization of azobenzene triggered by UV light. However, due to the limited penetration depth of UV light, only the surface azobenzene moieties undergo isomerization, resulting in a relatively small overall change in the measured modulus.

**Figure 4:**
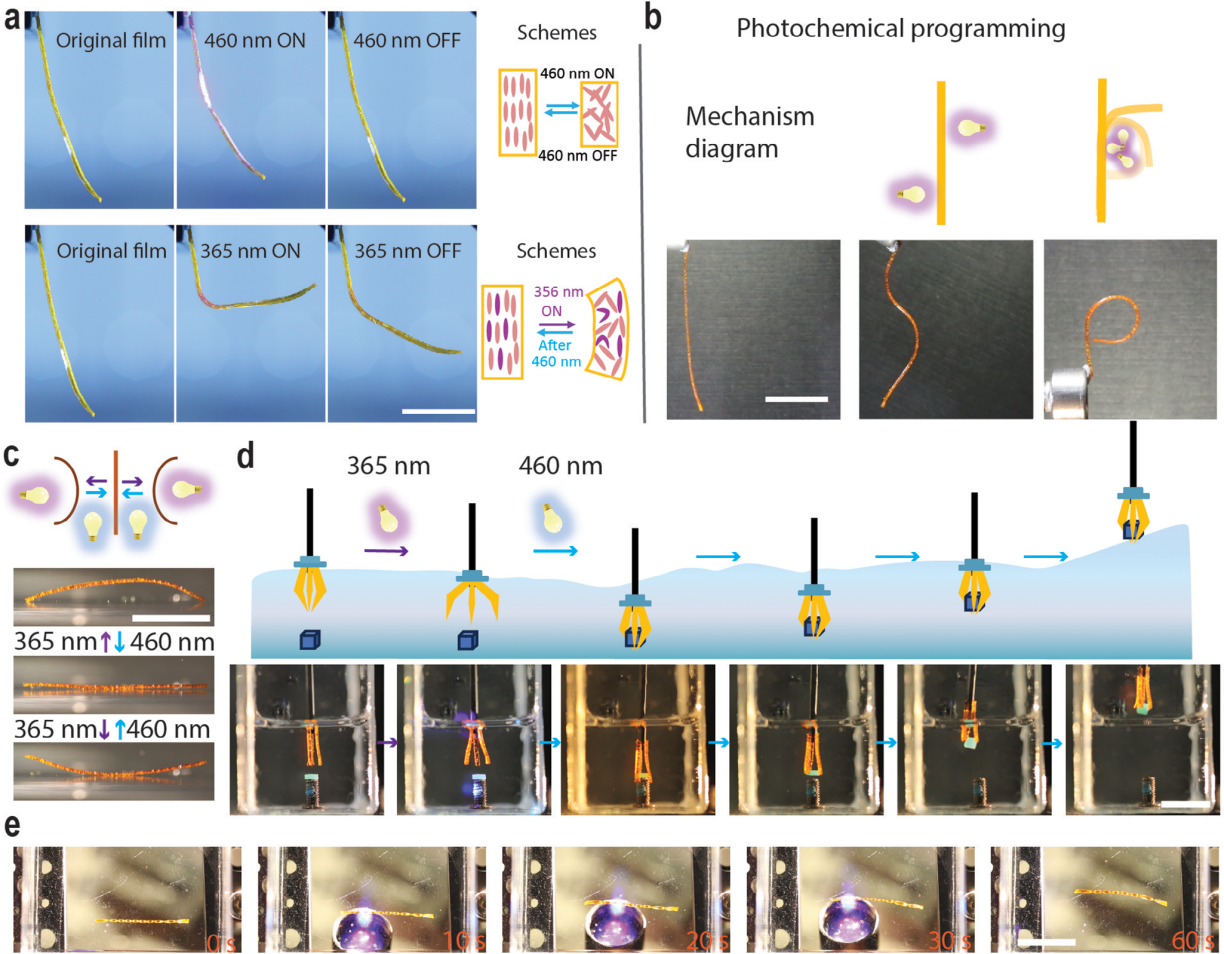
Programmable soft actuators. a) Left: Photographs of photothermal contraction (460 nm, 810 mW cm^−1^) and photochemical bending (365 nm, 390 mW cm^−2^) of LCE‐Azo 15%. Right: Schematic illustration of the corresponding actuation schemes. b) Photochemical shape programming of LCE‐Azo 15%. c) Schematic diagram and photographs illustrating underwater bending of LCE‐Azo 15% toward the light irradiation direction. d) Schematic diagram and photographs illustrating underwater gripping. The gripper is opened with UV light (365 nm, 390 mW cm^−2^) and fixed around the object with visible light (460 nm, 810 mW cm^−2^). e) Photographs of underwater self‐rolling of LCE‐Azo 15%. Actuator dimensions: 24 × 1 × 0.4 mm^3^. Light intensity: 365 nm, 390 mW cm^−2^. All scale bars: 1 cm.

The LCE‐Azo X% actuators combine i) distinct shape morphing via photochemical and photothermal actuation pathways (resulting in bending and contraction, respectively, as illustrated in **Figure** **4**a) and ii) shape programming enabled by the dynamic bonds. This allows for various deformations and multi‐mode robotic actuation, as exemplified in Figure 4 using LCE‐Azo 15%. The shape programming ability is illustrated in Figure S21 (Supporting Information), where samples are heated to 100 °C, deformed to a desired shape, and fixed upon cooling. After 3 days of equilibration, the samples are ready for use. The photochemically induced shape changes can be “locked” for extended periods, allowing for photochemical programming of the stretched, uniaxially aligned samples (Figure 4b; Video S3, Supporting Information). Figure S22 (Supporting Information) presents a twisted LCE strip that self‐rolls on a 100 °C hot plate and autonomously changes its direction of motion when encountering an obstacle. This occurs because the impacted part comes to a stop while the remaining sections continue to move, creating a rebound effect that changes the direction of motion.^[^ ^68^ ^]^ The strip also exhibits light tracking properties (Figure S23 and Video S4, Supporting Information), enabling movement steering with 365 nm illumination. When confined inside a tube, a stretched LCE strip can move under scanned illumination (Figure S24 and Video S5, Supporting Information). The motion arises from lateral expansion, which anchors one end of the strip to the tube wall while the free end moves, resulting in a gradual shift opposite to the light scanning direction.

In an aqueous environment, LCE‐Azo 15% bends toward the UV light source and reverts to its original shape under 460 nm irradiation (Figure 4c), the bending process stabilizes within tens of seconds, and the recovery follows shortly after. Visible‐light irradiation does not induce actuation due to the high thermal dissipation in water.^[^ ^69^ ^]^ The bending is moderate in the thick (480 µm after stretching) LCE strips due to limited light penetration depth, yet sufficient to operate underwater grippers. These grippers can open under 365 nm illumination and close around objects under 460 nm illumination, enabling grasping and manipulation (Figure 4d; Video S6, Supporting Information). The UV‐light‐induced self‐sustained rolling motion can also be attained underwater (Figure 4e; Video S7, Supporting Information). Although the rolling speed and distance are modest, this demonstrates the feasibility of photochemically driven, self‐sustained rollers in submersed conditions.

**Figure 5:**
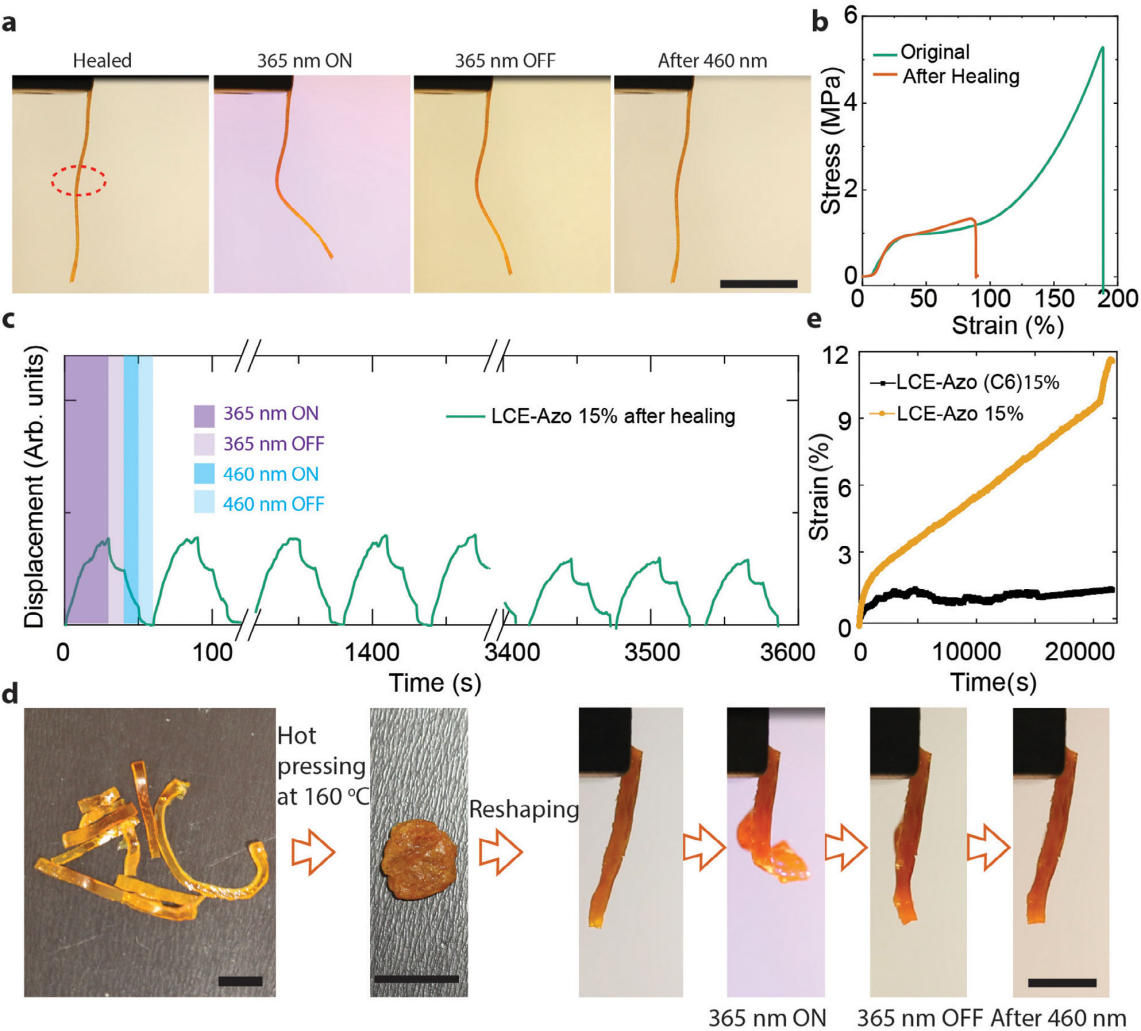
Reprocessing of LCE‐Azo 15%. a) Photographs of photochemical actuation (365 nm, 320 mW cm^−2^) after welding. Sample dimensions: 30 × 1.0 × 0.5 mm^3^. b) Tensile stress‐strain curves of the original and welded LCE‐Azo 15% strips. Welding was performed at 80 °C for 5 min. c) Reversible photochemical actuation of LCE‐Azo 15% after welding over 60 cycles excitation cycles (365 nm, 400 mW cm^−2^; 460 nm, 160 mW cm^−2^). d) Reprocessing of LCE‐Azo 15% through compression molding. The sample was heated to 160 °C, compressed, and subsequently reshaped. The reprocessed sample still maintained photochemical activity. e) Creep curves (measured at 80 °C) of LCE‐Azo (C6) 15% and LCE‐Azo 15%.

One of the key advantages of LCEs incorporating supramolecular or dynamic covalent bonds is their ability to “self‐repair” through bond exchange, enabling reprocessing and remolding.^[^ ^57^, ^58^, ^70^, ^71^, ^72^, ^73^ ^]^ This feature is also present in the samples studied in this work. In **Figure** **5**a, we show an LCE‐Azo 15% strip that was cut and then welded back together by placing the connected ends on an 80 °C hot plate for 5 min. Although the welding process decreased the tensile strength and fracture strain (Figure 5b), the sample retained its photochemical activity, both as a free‐standing strip (Figure 5a) and under an external load (Figure S25 and Video S8, Supporting Information). The welded sample exhibited excellent repeatability, maintaining photochemical performance even after 60 activation cycles, though with a gradual decrease in efficiency (Figure 5c; Figure S26 and Video S9, Supporting Information).

Figure 5d shows that a reprocessed sample, once stretched, retained a portion of its photochemical activity. With further optimization, this opens the door to developing recyclable halogen‐bonded LCEs with extended lifespan and improved durability. To emphasize the importance of combining disulfide and supramolecular XB crosslinks, the dynamic properties were studied using creep (25 °C) and stress relaxation (80 °C) experiments (Figure 5e; Figure S27, Supporting Information). When the disulfide bonds were replaced with static carbon–carbon bonds, the presence of halogen bonds alone resulted in an initial rapid strain release. In contrast, samples containing both disulfide and halogen bonds displayed an initial rapid creep phase due to halogen bonds, followed by a slower, sustained creep phase attributed to the dynamic disulfide bonds (Figure 5e). Additionally, we observed that increasing the temperature accelerates the bond exchange rate (Figure S27a, Supporting Information). The stress relaxation curves further support this: with only halogen bonds, the material quickly relaxes to a stable state, whereas with disulfide bonds, the relaxation involves a fast initial release followed by a slower descent to equilibrium (Figure S27b, Supporting Information). This enables reprocessing of the LCE‐Azo 15%, as demonstrated by cutting a larger sample into small fragments and compressing them at a high temperature (160 °C) to reform the desired material (Figure 5d). Raman spectroscopy confirmed the presence of the halogen bonds after compression molding (Figure S28, Supporting Information).

## 3 Conclusion

We introduce a multi‐mode liquid crystal elastomer (LCE) actuator capable of reversible shape morphing, shape programming, and reprocessing, achieved through the synergistic use of Aza–Michael addition reaction and dynamic bonds. This approach eliminates the need for photoinitiators and enables precise control over azobenzene content, a key factor influencing the photochemical actuation performance of the LCE. The dynamic bonds facilitate shape programming and recycling, allowing the material to be reused multiple times. Our design leverages both supramolecular halogen bonds and dynamic disulfide bonds which together provide the needed mechanical and dynamic properties for efficient and reprogrammable actuation. We comprehensively characterize the material properties and demonstrate proof‐of‐concept robotic motions, including self‐sustained rolling propelled by photothermal and photochemical gradients, and showcasing potential applications in underwater robotics. Our results bolster the applications of dynamic, supramolecular LCEs for multi‐functional and photochemically responsive actuators.

## Conflict of Interest

The authors declare no conflict of interest.

## Author Contributions

H.G. and A.P. conceived the material concept; H.G. conducted the experiments including synthesis and characterization and conceived the robot concept; H.G. and R.N. carried out Raman and UV spectrum analysis; K.K. and R.N. carried out cis lifetime analysis. C.L. and H.G. carried out WAXS experiments and analysis of the data. H.G. carried out the DMA experiment and analysis with the help of E.T. The project was planned jointly by H.G. and A.P. H.G. and A.P. wrote the manuscript. All authors contributed to the discussion and polishing of the manuscript.

## Supporting information



Supporting Information

Supplemental Video 1

Supplemental Video 2

Supplemental Video 3

Supplemental Video 4

Supplemental Video 5

Supplemental Video 6

Supplemental Video 7

Supplemental Video 8

Supplemental Video 9

## Data Availability

The data that support the findings of this study are available from the corresponding author upon reasonable request.
