# Supplementary material for: Halogen‐Bonded Liquid Crystal Elastomers as Initiator‐Free Photochemical Actuators
Source: Adv Mater. 2025 May 28;37(33):2504551. doi: 10.1002/adma.202504551 (PMC12369687; doi:10.1002/adma.202504551)
Supplement: Supplementary file 1 — Supporting Information [file ADMA-37-2504551-s007.pdf]

ADVANCED MATERIALS

Supporting Information

for *Adv. Mater.*, DOI 10.1002/adma.202504551

Halogen-Bonded Liquid Crystal Elastomers as Initiator-Free Photochemical Actuators

Hongshuang Guo, Roshan Nasare, Chen Liang, Kim Kuntze, Eugene M. Terentjev and Arri Priimagi**

Supporting information for

Halogen-bonded Liquid Crystal Elastomers as Initiator-free Photochemical Actuators

Hongshuang Guo,[1*] Roshan Nasare,[1] Chen Liang,[2] Kim Kuntze,[1] Eugene M. Terentjev,[3] and Arri Priimagi[1*]

[1] Smart Photonic Materials, Faculty of Engineering and Natural Sciences, Tampere University, P.O. Box 541, FI-33101 Tampere, Finland.

[2] Department of Applied Physics, Aalto University, P.O. Box 15100, FI 02150 Espoo, Finland.

[3] Cavendish Laboratory University of Cambridge, J. J. Thomson Avenue, Cambridge CB3 0HE, UK

Correspondence to: hongshuang.guo@tuni.fi, arri.priimagi@tuni.fi.

This PDF file includes:

Materials and Methods
Figures S1 to S28
Captions for Movies S1-S9

Additional supplementary material for this manuscript includes:

Movies S1-S9

Materials and Methods

Materials in brief. 1,4-Bis-[4-(6-acryloyloxyhexyloxy)benzoyloxy]-2-methylbenzene (99%, RM82) and 4,4'-Bis(6-acryloyloxyhexyloxy)azobenzene (Azo) were purchased from SYNTHON Chemicals GmbH & Co. 1-amino-3-(dimethylamino)propane (**A**) and cystamine dihydrochloride were purchased from TCI. Cystamine was obtained by treating the cystamine dihydrochloride with NaOH and extracting it from dichloromethane. 1,4-Diiodotetrafluorobenzene (**D**) was purchased from Fluorochem Ltd. All reagents and chemicals were used as received without further purification.

Film preparation. LCE cells were prepared by gluing two glass substrates coated with polyvinyl alcohol (PVA, 5% water solution, 4000 RPM for 1 min and baked at 100 ºC for 10 min). Microspheres with a diameter of 550 μm (Thermo Scientific) were used as spacers to determine the LCE film thickness. Taking LCE-Azo 15% as an example, the liquid crystal mixture was prepared by combining 0.85 mmol RM82, 0.15 mmol Azo, 0.3 mmol cystamine, 0.4 mmol **A**, and 0.2 mmol **D** at 100 ºC until all components melted into a clear liquid. The mixtures were infiltrated into the cells via capillary action at 100 ºC and then cooled down to 60 ºC. The cells were placed in an oven to allow the Aza-Michael addition reaction for oligomerization to proceed for 4 hours at 60 ºC, followed by heating to 90 ºC to continue the reaction for another 20 hours. The polymerized cells were opened using a blade, and strips with desired dimensions were cut from the film. To align the initially disordered LCE-Azo X%, the samples were heated to 100 °C, uniaxially stretched (typically by 100 %), and subsequently cooled to room temperature. The aligned LCEs were kept at room temperature for at least 72 h before characterization and testing during which time, due to stress-induced contraction, the sample length stabilized to ca. 150 % of the original length before stretching.

Actuation measurements. Thermal actuation measurements were performed by placing aligned samples on a black anodized aluminium sheet on a hotplate. The samples were heated from 30

to 110 °C by increasing the temperature at 10 °C intervals. The reversibility of the actuation was ensured by monitoring the shape changes upon both heating and cooling. All samples were subjected to a full heating and cooling cycle to erase the thermal history before the actuation measurement. The photochemical bending test involves suspending the sample vertically in the air, and then illuminating it with UV light of varying intensities. Subsequently, photographs are taken to estimate the bending angle.

Preparation of light-driven roller and underwater gripper. The rolling LCE was just a single monodomain LCE-Azo 15% strip that was shape-programmed to obtain a twisted configuration. The stretched and twisted LCE-Azo 15% strip was placed on a hot plate or illuminated (*i.e.*, photothermally heated). The underwater gripper consists of three LCE-Azo 15% strips, each 1 cm in length, adhered to the top of a metal cylinder, forming a claw-like structure. It can be photochemically actuated underwater to grab and manipulate objects.

Welding and reprocessing. The methodology for assessing the self-healing capacity involves sectioning the LCE-Azo 15% strip into two segments, realigning the cut pieces under mild temperature stimulation on a hot plate, and subsequently subjecting the healed samples to mechanical testing. The healing efficiency (%) denotes the ratio of the recuperated toughness to the initial toughness, accounting for the restitution of both stress and strain. The recyclable samples are obtained by cutting the samples into multiple pieces, followed by hot pressing at high temperatures and compressed into the desired shape.

Material characterization. Raman spectra were obtained using Renishaw InVia Qontor Raman microscope equipped with a 785 nm laser. The spectra were collected in the range of 110-1400 cm^{-1} (Fig. S4).

DSC measurements were performed with a NETZSCH DSC 214 *polyma* instrument at a heating/cooling rate of 10 °C min^{-1}. The measurements were performed using a 7–12 mg

sample under a 1 bar nitrogen atmosphere (flow rate of 20 mL min^{-1}) at the temperature range between -50 – 150 °C.

Tensile tests were performed by using a homemade tensile tester. The dimensions of the rectangular film were measured with a digital calliper. The tensile speed was set at 5 mm min^{-1}. Each measurement was repeated three times.

The FTIR spectra of the LCE-Azo X% were recorded using the INVENIO FT-IR Spectrometer Platform.

DMA measurements were conducted using TA Instruments DMA 850 in a thin film tension mode within a temperature-controlled chamber. The dimension of the rectangular film was approximately 16 × 2 × 0.5 mm^3, as measured with a digital calliper. The sample was measured at 1 Hz frequency and 0.1 % stain with 0.01 N preload force (force tracking at 125 %). The sample was heated from -50°C to 150°C with a heating rate of 5 °C min. For stress relaxation experiment, the temperature is 80°C, strain is 3%. For creep experiment, the temperature is 30°C, stress is 0.02N.

UV-visible absorption spectra and *cis*-lifetime were recorded with an Agilent Cary 60 spectrophotometer, from films with a thickness of 1 μm.

The alignment of the monodomain sample was characterized by a polarized optical microscope (Zeiss, AXIO Scope.A1) by imaging the samples at 0 and 45° angles between the polarizer/analyzer axes.

Wide-angle (WAXS) X-ray scattering measurements were conducted using a Xenocs Xeuss 3.0 SAXS/WAXS system (Xenocs SAS, Grenoble, France) equipped with an area detector (Eiger2 R 1M, Dectris AG, Switzerland). This system comprises two microfocus X-ray sources employing Cu or Mo targets. The X-ray beams were collimated with a multilayer mirror,

generating a parallel beam with a nominal wavelength of either 0.711 Å (combined Mo K-α_1 and Mo K-α_2 characteristic radiation) for temperature dependent measurement or 1.542 Å (combined Cu K-α_1 and Cu K-α_2 characteristic radiation) used for room-temperature measurements. The X-ray source operated at an operating voltage of 50 kV and a current of 1.0 mA (Mo target) or 0.6 mA (Cu target). The beam size at the sample position was set to 0.7 mm × 0.7 mm. For WAXS measurements, the distance between the samples and detector was 150 mm for the temperature dependent measurement and 55 mm for room temperature measurements. The calibration of the sample-to-detector distance was performed using a LaB_6 standard sample. The measurements were conducted in a vacuum chamber. The orientation parameter (*S*) is calculated based on the Hermans-stein orientation distribution function, where the I(θ) is the intensity at an azimuthal angle of (θ).

$$S = \frac{3\langle cos^2\theta\rangle - 1}{2}$$

$$\langle cos^2\theta\rangle = \frac{\int_0^{\pi/2} I(\theta) sin\theta cos^2\theta d\theta}{I(\theta) sin\theta d\theta}$$

All photographs and supplementary movies were taken with a Canon 5D Mark III camera equipped with a 100 mm lens. Surface temperature changes were recorded with an infrared camera (FLIR T420BX) equipped with a close-up (2×) lens. CoolLED pE-4000 was used as the UV light source (365 nm) for robot walking demo and light actuation experiments. Kinovea program was used to monitor the motion of the trajectory of the vertex, yielding distance and time data at 1/30 s time intervals as coordinates for the x- and y-axis.

Supplementary Figures

a

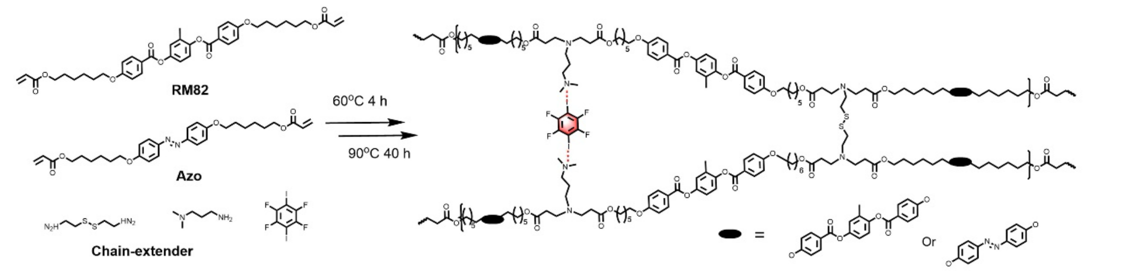

b

	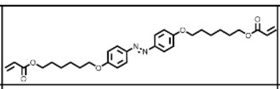	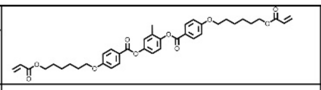	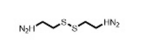	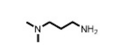	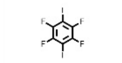
LCE Azo 5%	0.05	0.95	0.3	0.4	0.2
LCE Azo 15%	0.15	0.85	0.3	0.4	0.2
LCE Azo 30%	0.3	0.7	0.3	0.4	0.2
LCE Azo 50%	0.5	0.5	0.3	0.4	0.2
LCE Azo 100%	1	0	0.3	0.4	0.2
LCE Azo 0%	0	1	0.3	0.4	0.2

Figure S1. (a) The chemical composition and preparation process of LCE-Azo X% to form a corresponding supramolecular network, and (b) the molar ratios of the compounds used for each mixture.

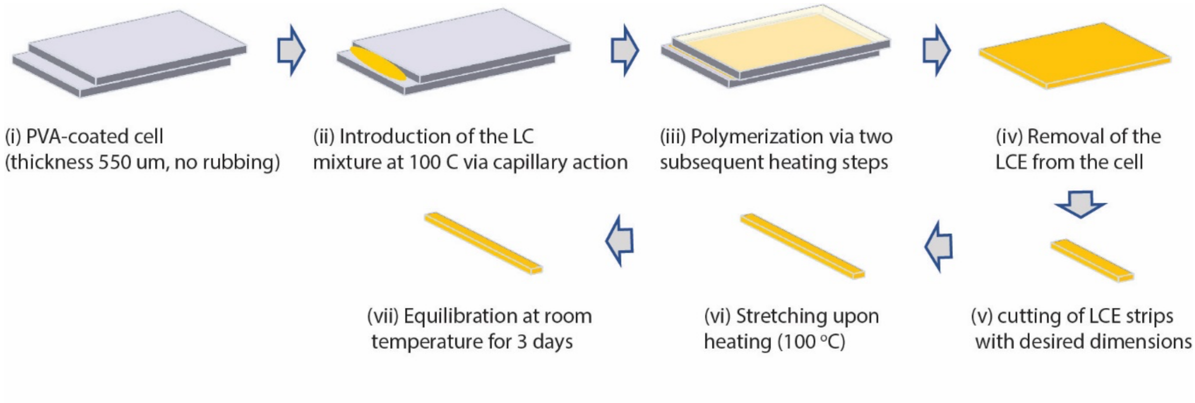

Figure S2. Schematic illustration of the preparation process of LCE-Azo X% strips.

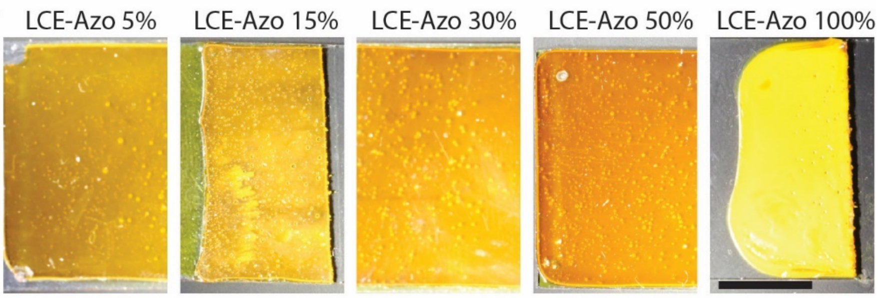

Figure S3. Photographs of LCE-Azo X%. Scale bar: 1 cm.

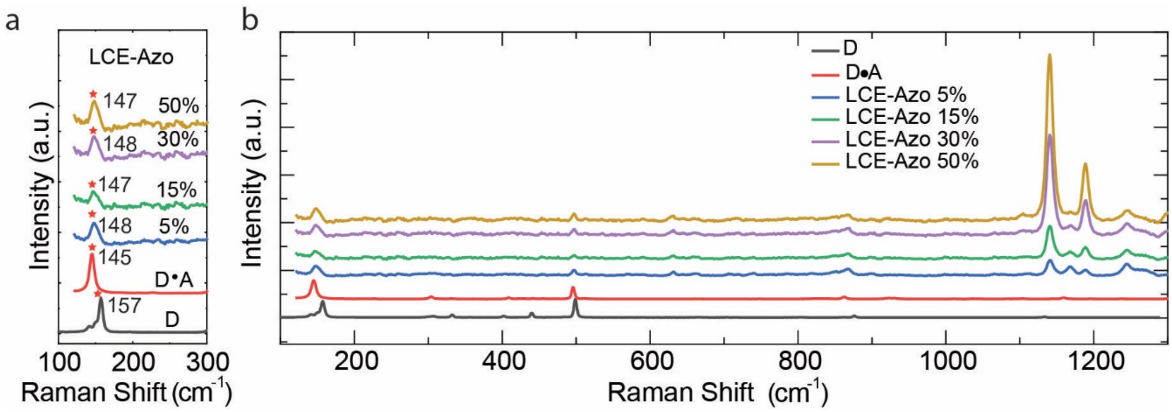

Figure S4. (a) Raman spectra of **D**, **D·A** and LCE-Azo X%, highlighting the redshift of the C–I stretching band due to XB formation. (b) Stacked Raman spectra of **D**, **D·A** and LCE-Azo X%.

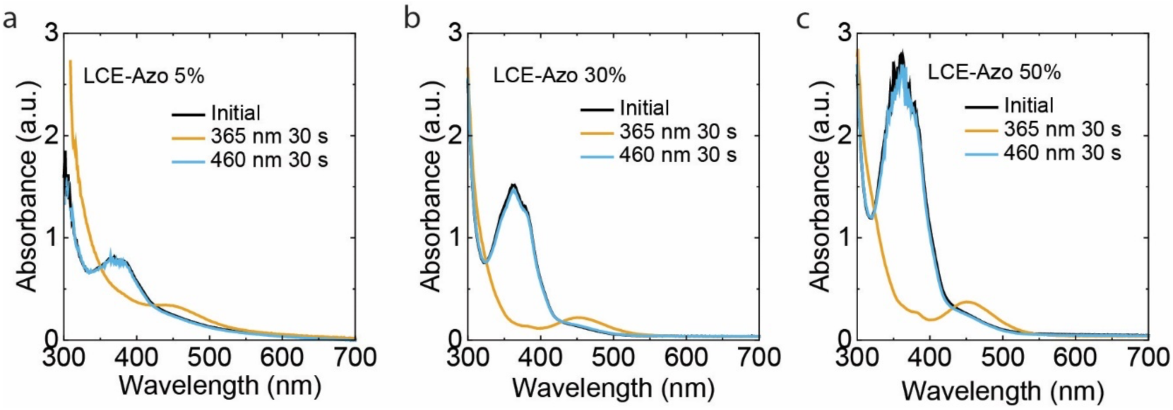

Figure S5. (a-c) The π–π* and n–π* band of Azo can be used to monitor the photoisomerization process, as shown for LCE-Azo 5%, LCE-Azo 30% and LCE-Azo 50% under subsequent 365 nm (35 mW cm^{-2}, 1 min) and 460 nm (45 mW cm^{-2}, 1 min) irradiation.

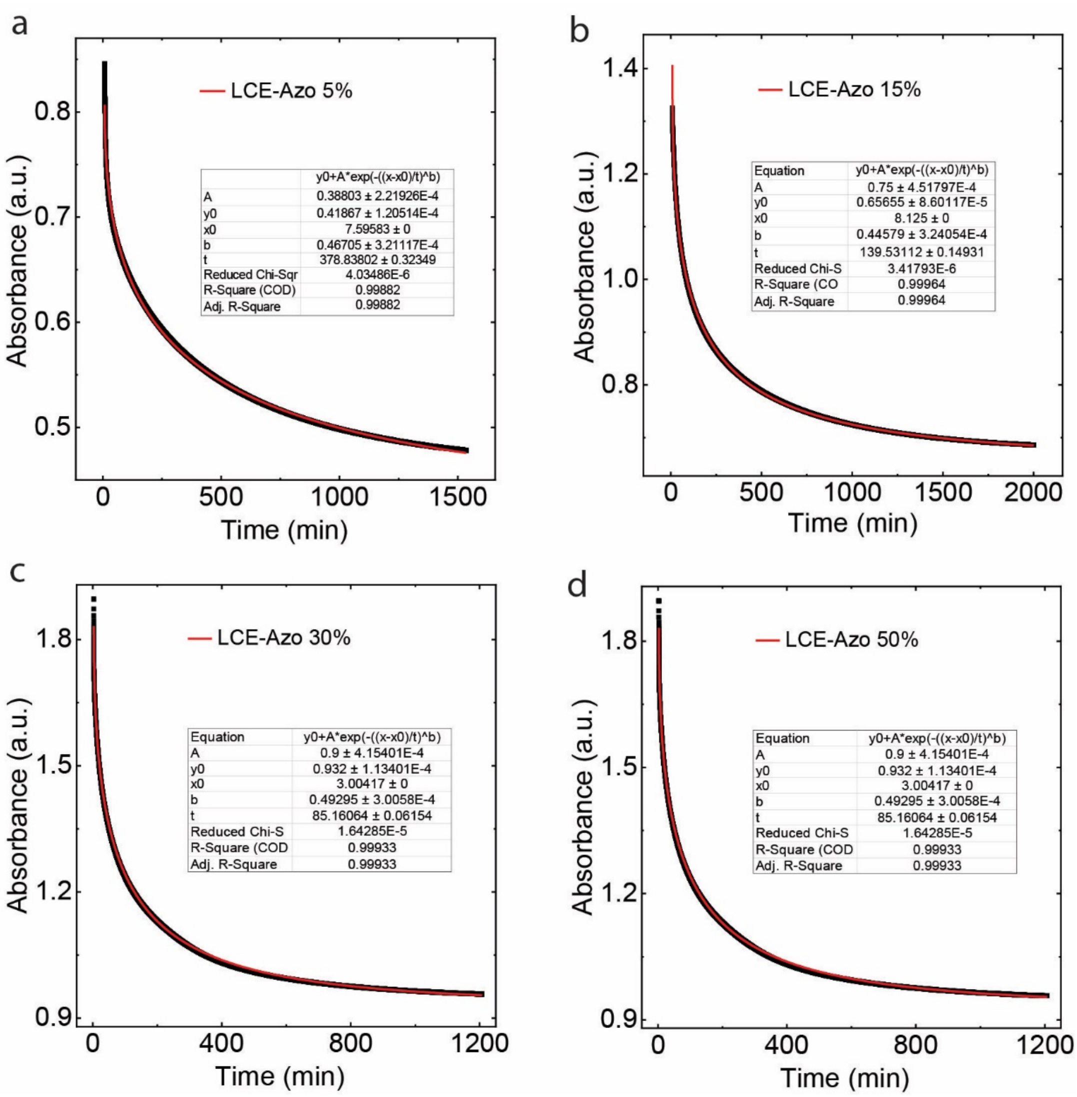

Figure S6. Thermal *cis–trans* relaxation and corresponding exponential fits to deduce the *cis* half-lives of (a) LCE-Azo 5% at 470 nm, (b) LCE-Azo 15% at 470 nm, (c) LCE-Azo 30% at 500 nm, and (d) LCE-Azo 50% at 500 nm.

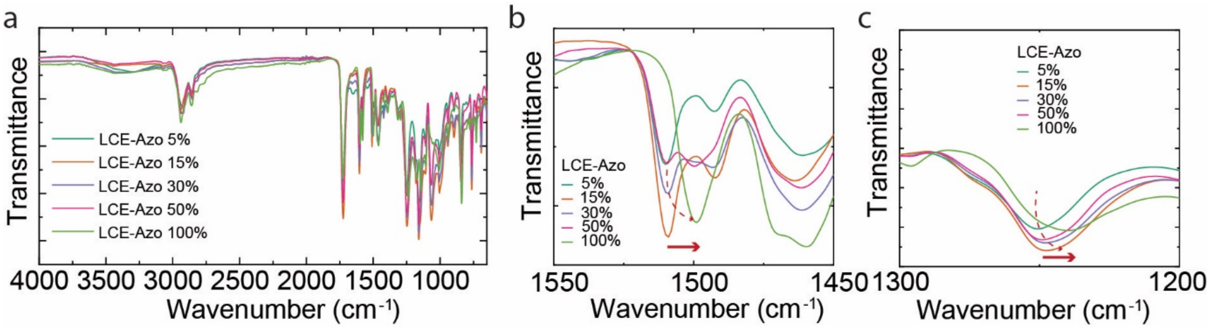

Figure S7. (a) FTIR spectra of the LCE-Azo X%, (b) from 1550-1450 cm^{-1} and (c) 1300-1200 cm^{-1}.

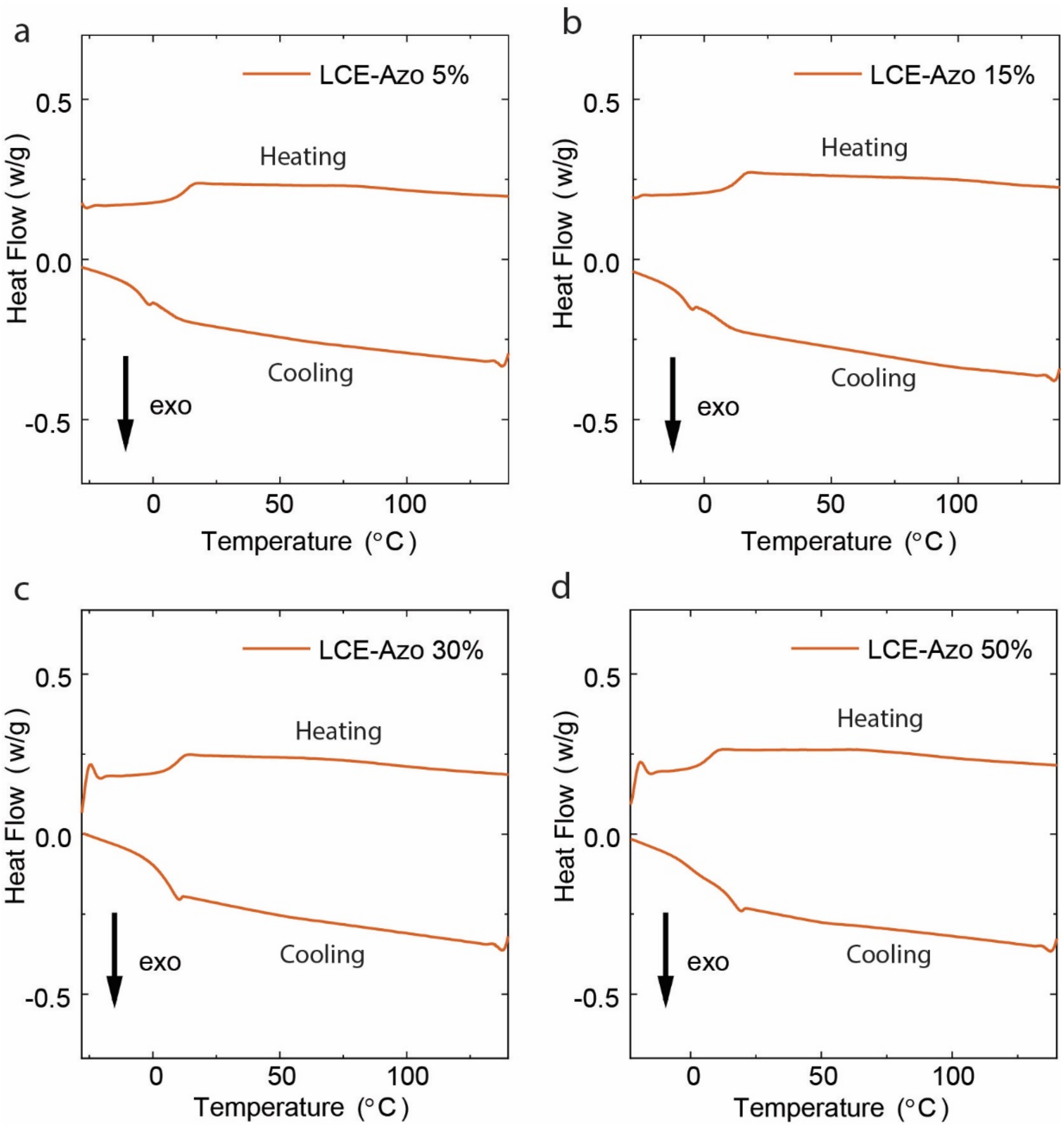

Figure S8. DSC curves of (a) LCE-Azo 5%, (b) LCE-Azo 15%, (c) LCE-Azo 30%, (d) LCE-Azo 50% upon second cooling and heating cycle. Heating/cooling rate:10 ºC/min.

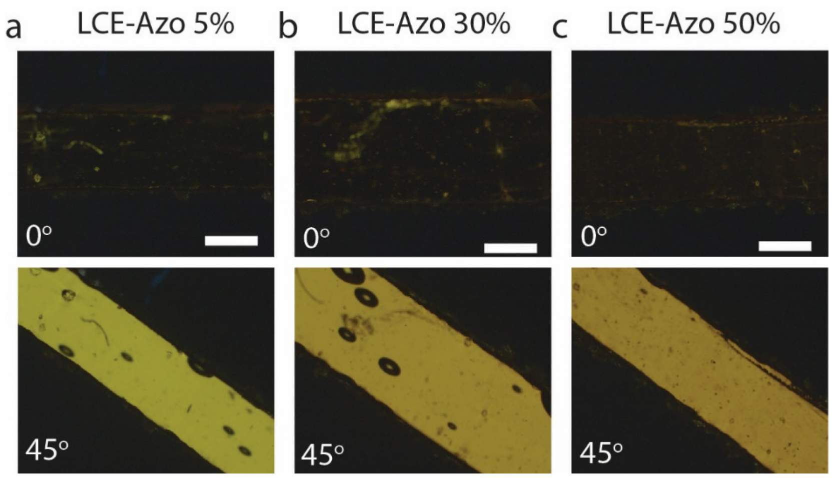

Figure S9. Polarized optical micrographs of the LCE-Azo X% after stretching, taken at 0° (top) and 45° (bottom) angles between the molecular director and the polarizer/analyzer, attesting uniaxial molecular alignment. (a) LCE-Azo 5%, (b) LCE-Azo 30%, (c) LCE-Azo 50%. Scale bars: 1 mm.

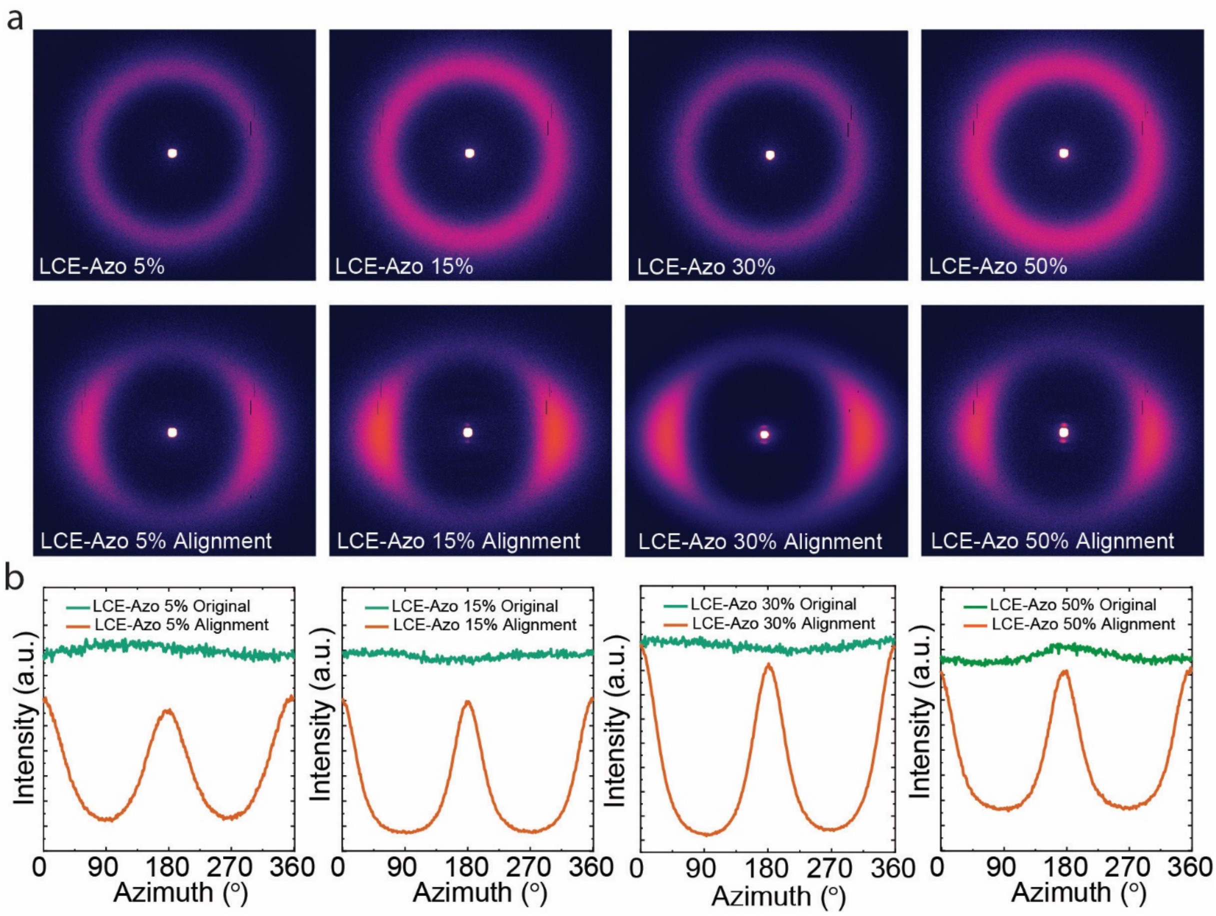

Figure S10. (a) 2D WAXS patterns of LCE-Azo X% before (top) and after (bottom) stretching and (b) corresponding 1D azimuthal scan profiles.

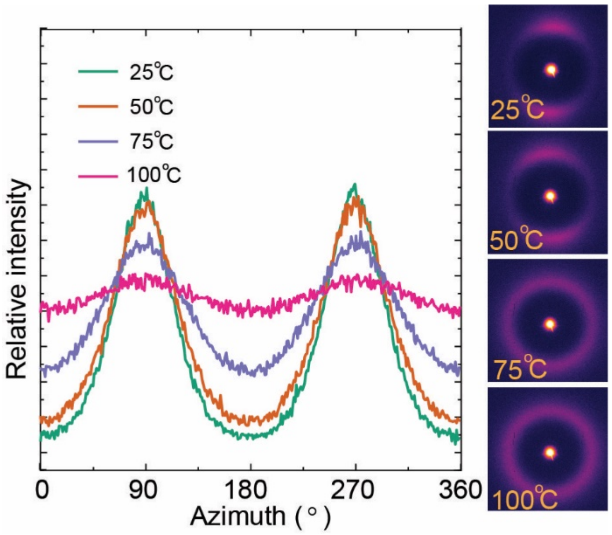

Figure S11. WAXS patterns of the aligned LCE-Azo 15% strip at different temperatures (right) and corresponding azimuthal profiles (left).

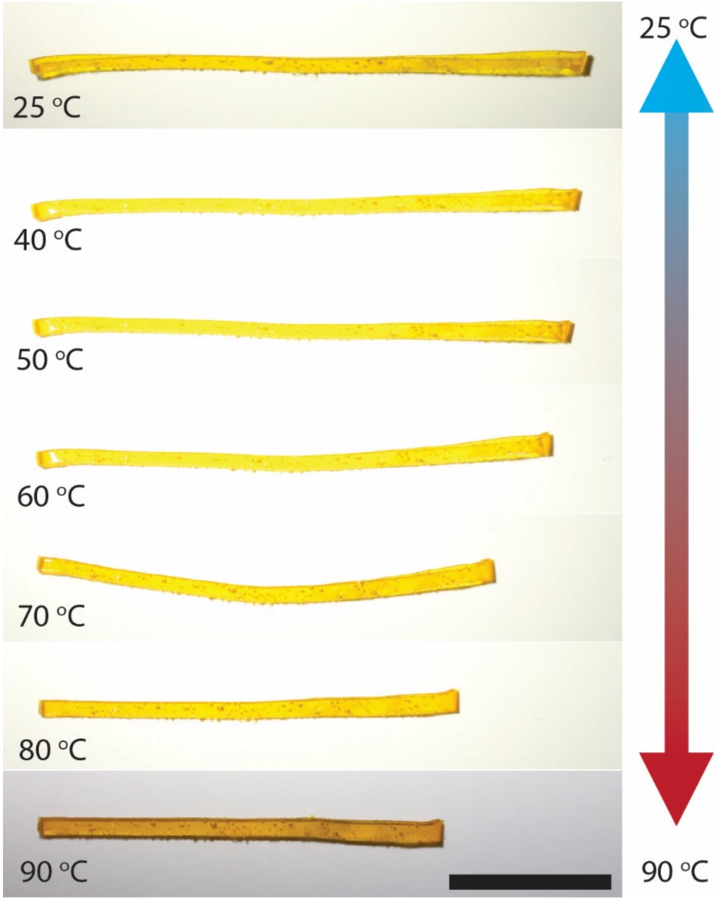

Figure S12. Photographs of thermal actuation of LCE-Azo 30% strip upon heating from 25 to 90 °C. Scale bar: 1 cm.

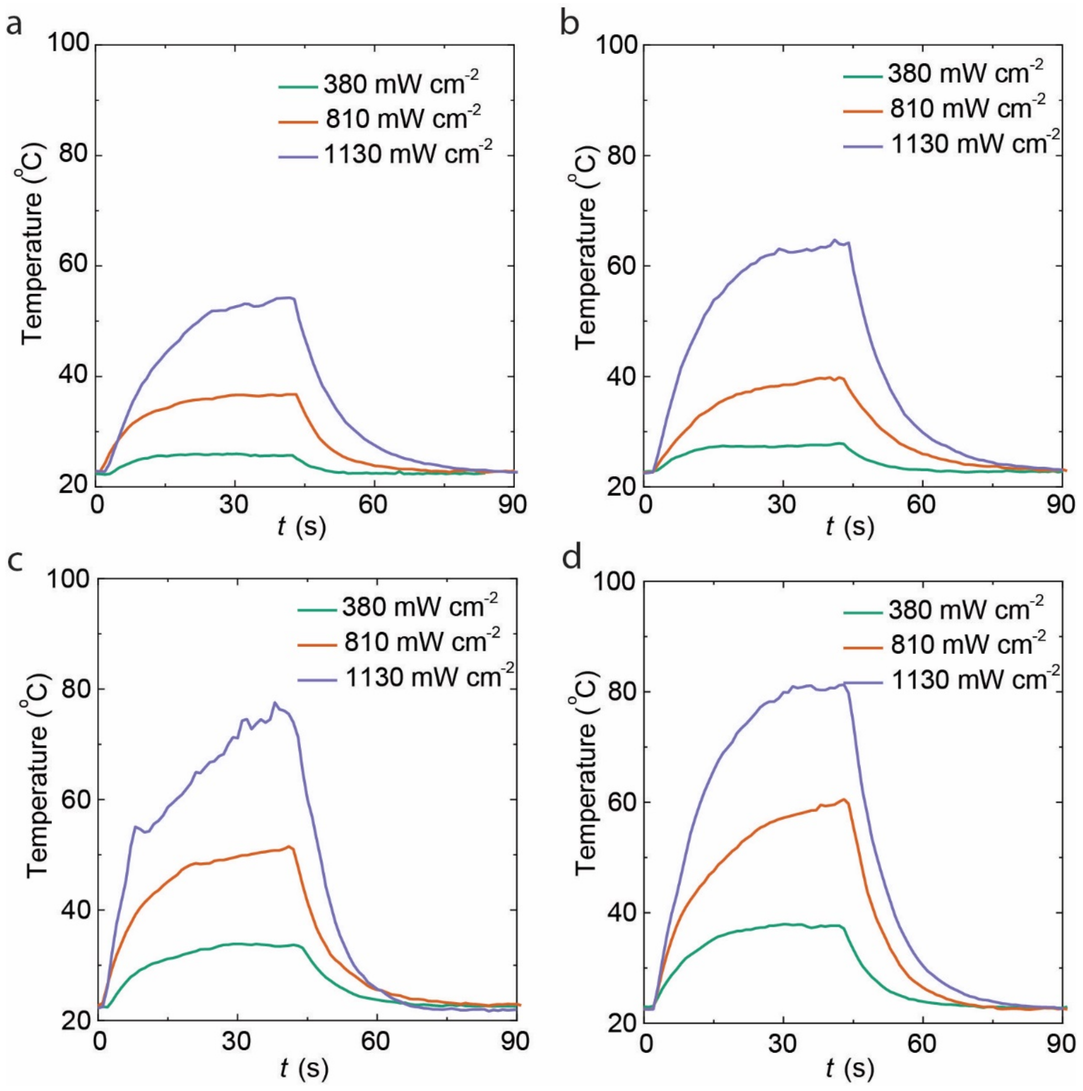

Figure S13. Photothermal temperature increase of LCE-Azo X% under 460 nm illumination with different intensities. (a) LCE-Azo 5%, (b) LCE-Azo 15%, (c) LCE-Azo 30% and (d) LCE-Azo 50%.

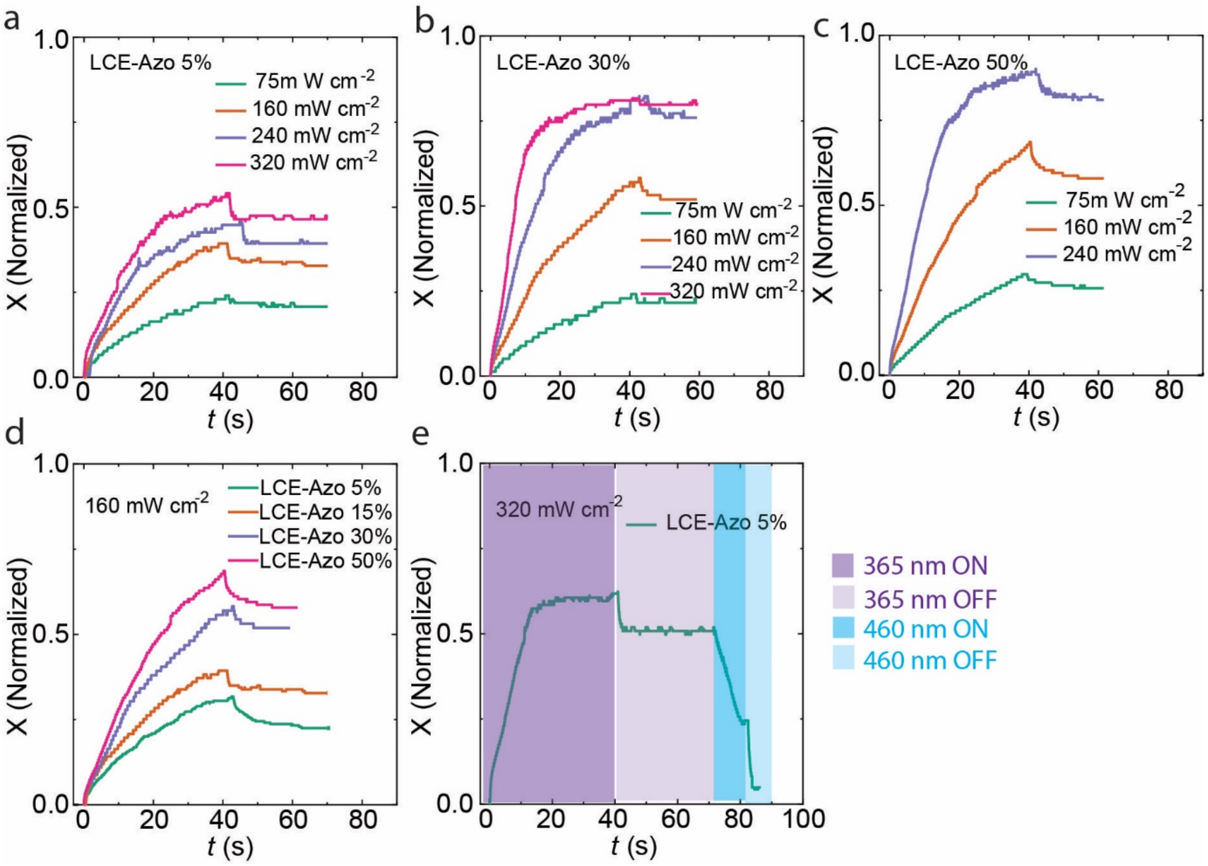

Figure S14. Bending kinetics of LCE-Azo X% under 365 nm irradiation with different intensities. The length of the X-directional movement of the tracking vertex is divided by the length of the strip itself. (a) LCE-Azo 5%, (b) LCE-Azo 30%, (c) LCE-Azo 50%. (d) Bending of the LCE-Azo X% under 365 nm illumination (160 mW cm^{-2}). The length of X-directional movement of the tracking vertex is divided by the length of the strip itself. (e) Photochemical deformation of LCE-Azo 5% strip.

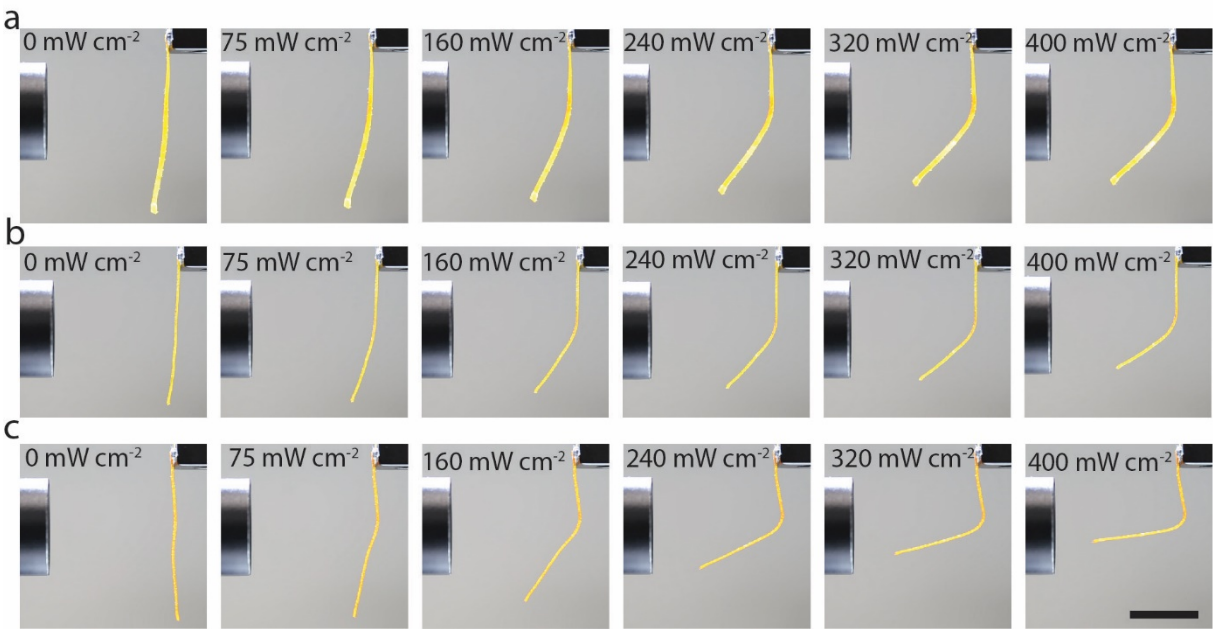

Figure S15. Photographs of photochemical deformation of LCE-Azo X% under different light intensities. (a) LCE-Azo 5%, (b) LCE-Azo 15% and (c) LCE-Azo 50%. Scale bar: 1 cm.

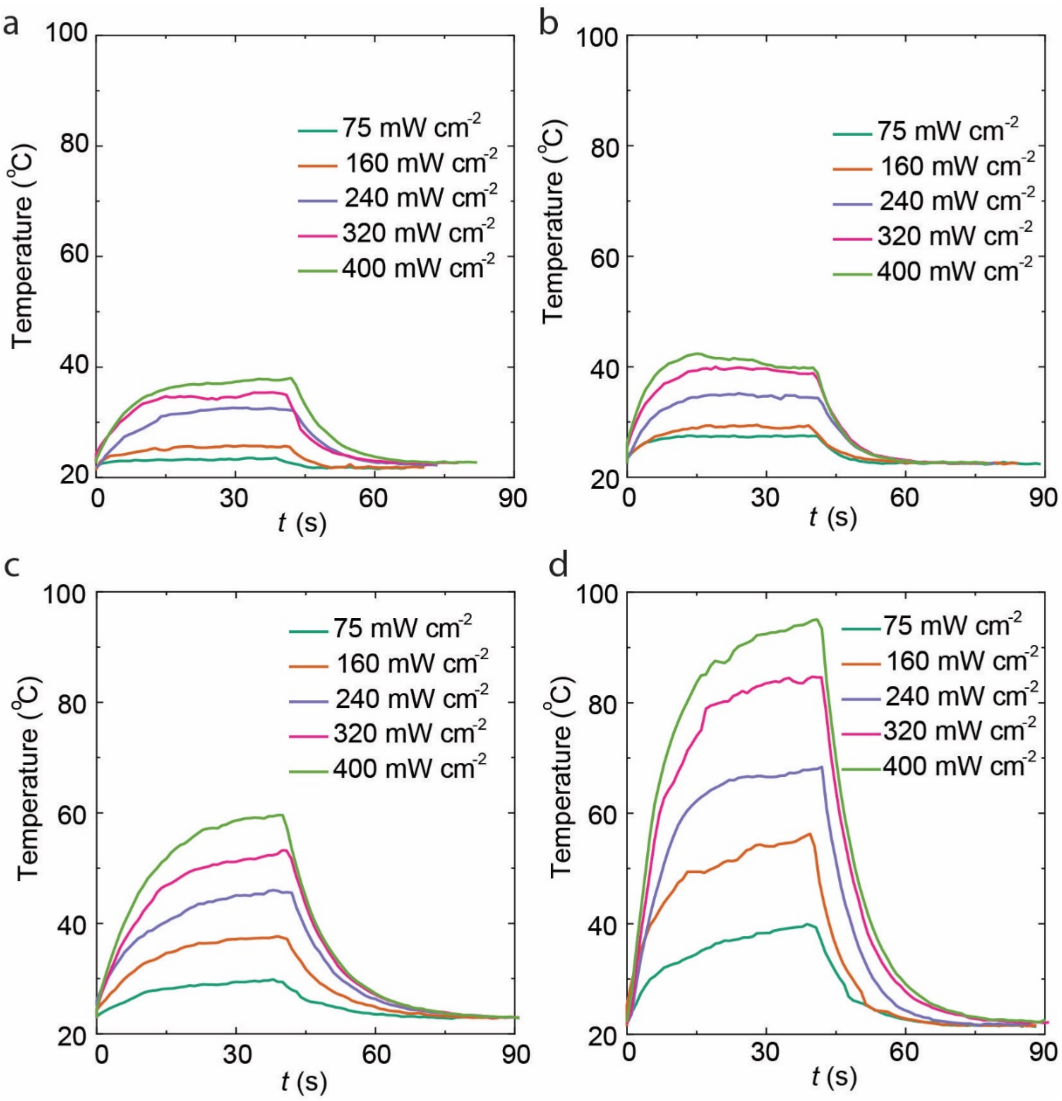

Figure S16. Kinetics of photothermally induced temperature increase of LCE-Azo X% strips under illumination with different intensities (365 nm). (a) LCE-Azo 5%, (b) LCE-Azo 15%,(c) LCE-Azo 30% and (d) LCE-Azo 50%.

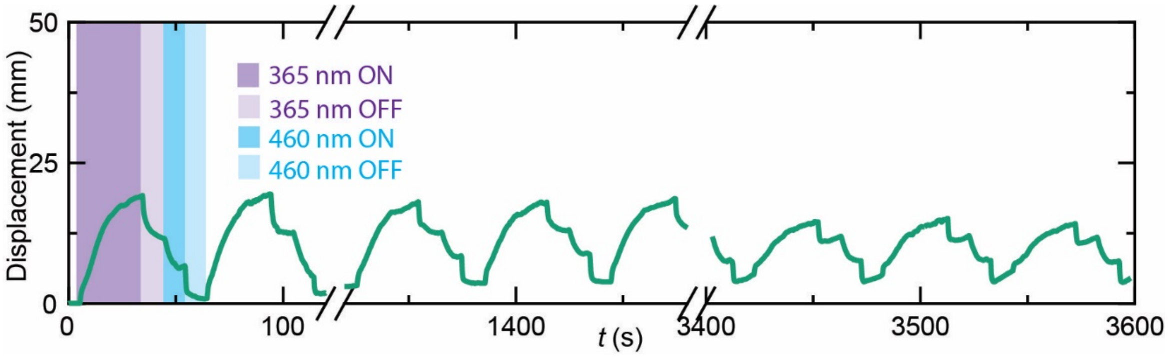

Figure S17. Photochemical deformation of LCE-Azo 15% strip upon 365 nm ON-365 nm OFF-460 nm ON-460 nm OFF light excitation for 60 cycles. Light excitation: 365 nm, 400 mW cm^{-2}, 460 nm, 160 mW cm^{-2}.

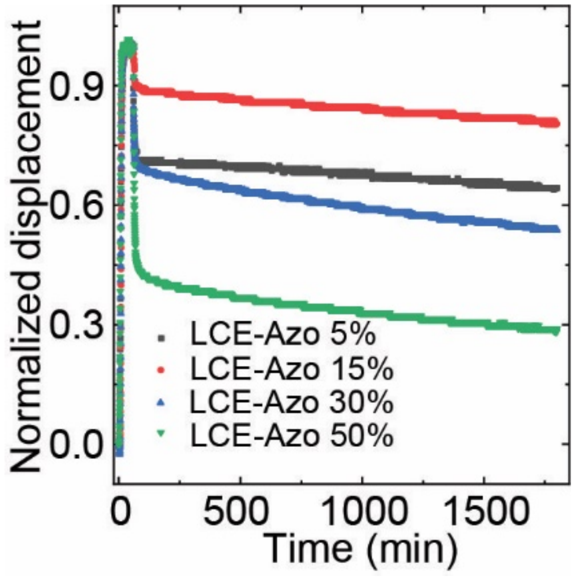

Figure S18. Tip displacement of LCE-Azo X% strip in the air during/after UV 365 nm (320 mW cm^{-2}) illumination.

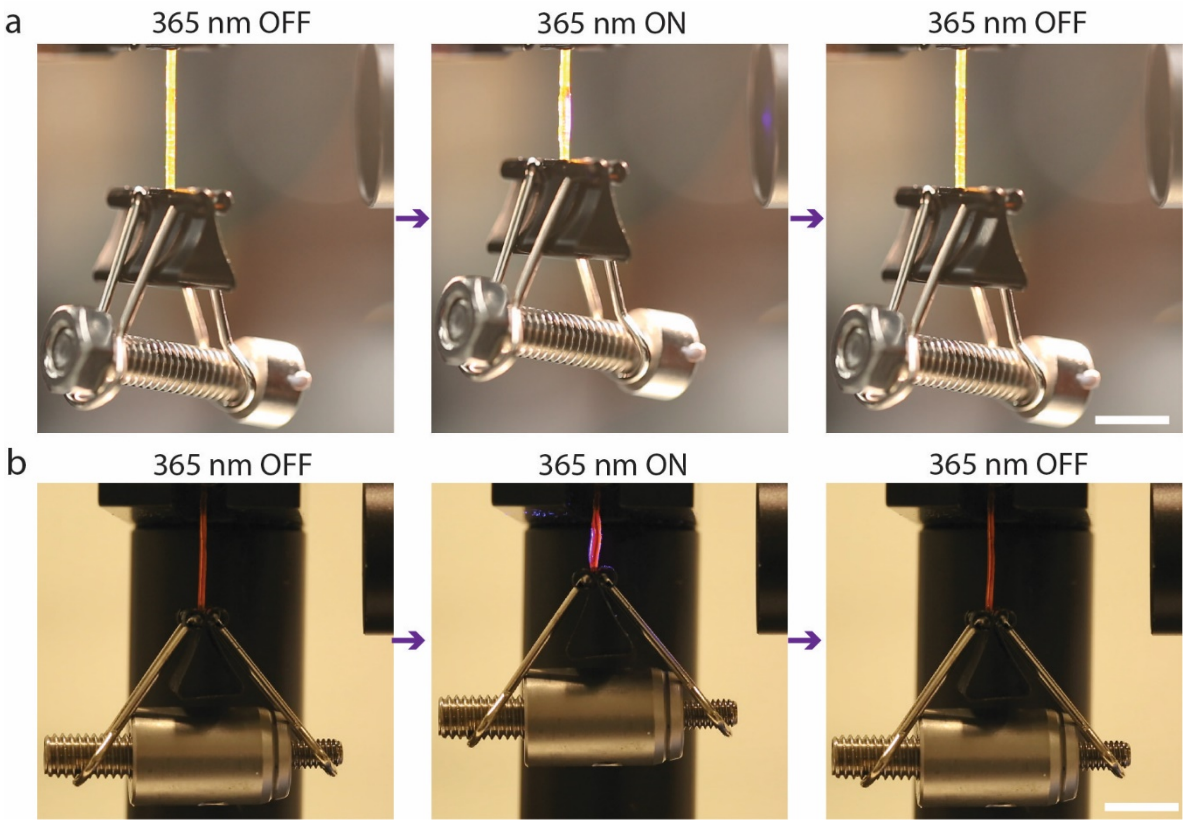

Figure S19. LCE-Azo 15% strip lifting heavy objects upon UV illumination. (a) 10 g, and (b) 50 g object lifted by LCE-Azo 15% strip weighing 0.03 g. Illumination: 365nm, 320 mW cm^{-2}. Scale bars: 1 cm.

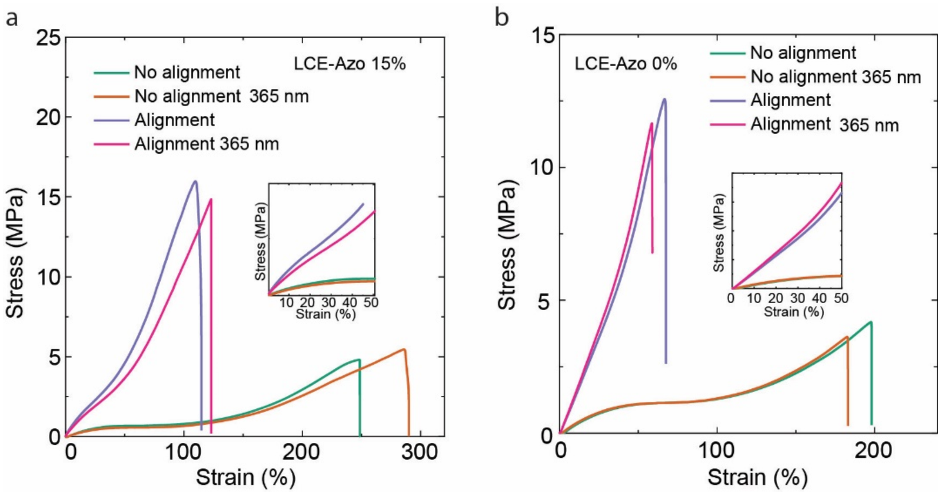

Figure S20. Stress–strain curves of the samples before and after UV irradiation along the alignment direction: (a) LCE Azo 15%, (b) LCE Azo 0%. The UV light intensity was 280 mW cm^{-2}, with an exposure time of 1 min per side.

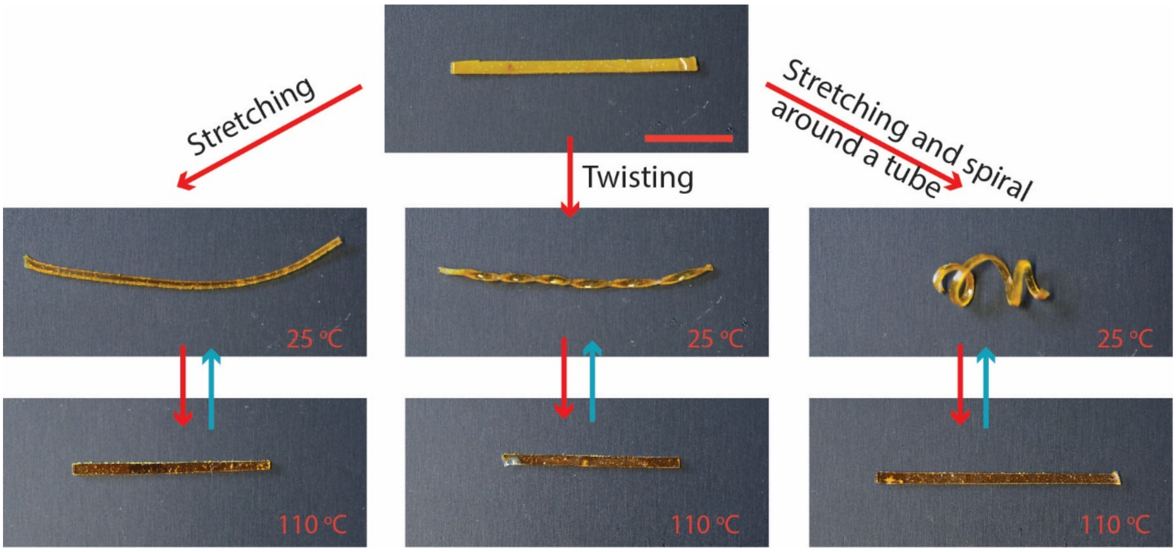

Figure S21. Photographs of shape-programmed LCE-Azo 15% strips. The programming was done at 100 °C, after which the shapes could be reversibly actuated. Scale bar: 1 cm.

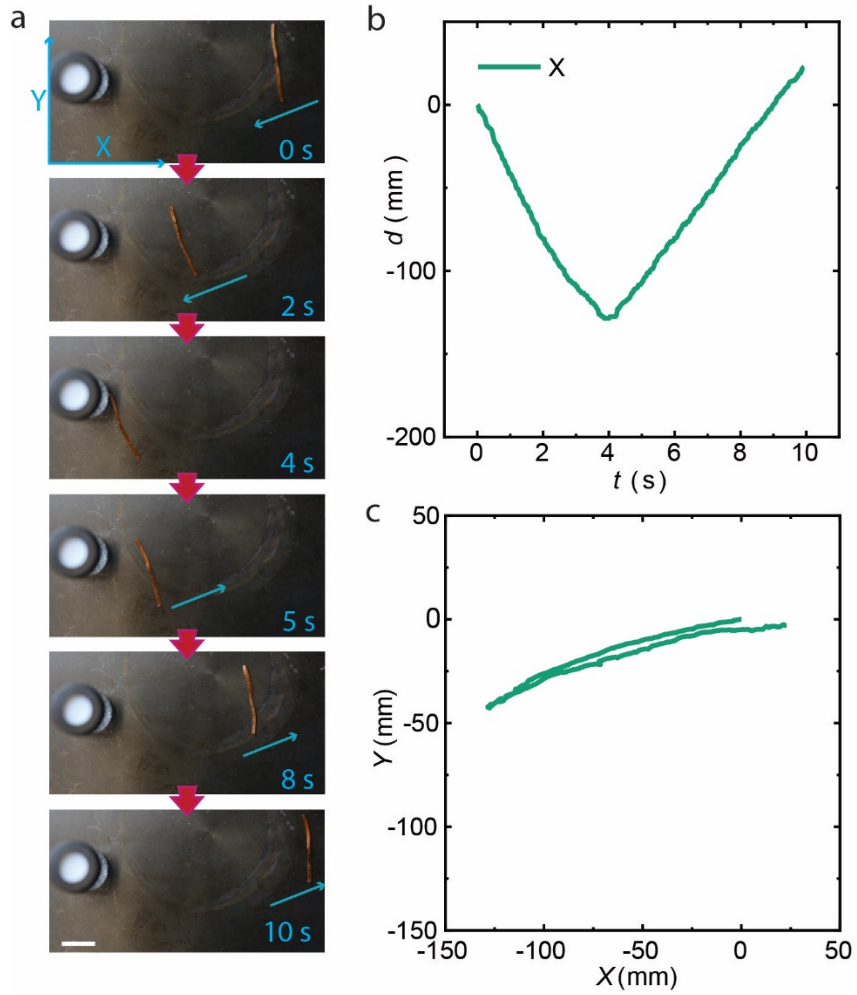

Figure S22. (a) Photographs of the self-rolling LCE-Azo 15% strip on a 100 °C hot plate, and autonomous direction reversal upon meeting an obstacle (a glass vial). Sample dimensions: 30×1.0×0.5 mm^3. (b) Heat-fueled rolling distance of the LCE-Azo 15% programmed construct. (b) Simply tracking the movement path along the X-axis, and (c) the motion trajectory in the X, and Y plane. X and Y directions were defined in (a). Scale bar: 1 cm.

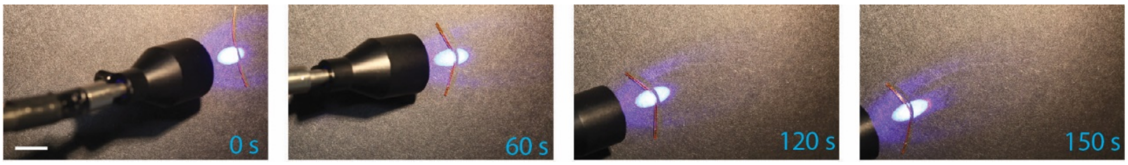

Figure S23. Photographs of the light-steered rolling of LCE-Azo 15% strip. Irradiation conditions: 365 nm, 390 mW cm^{-2}. Sample dimensions: 30×1.0×0.5 mm^3. Scale bar: 1 cm.

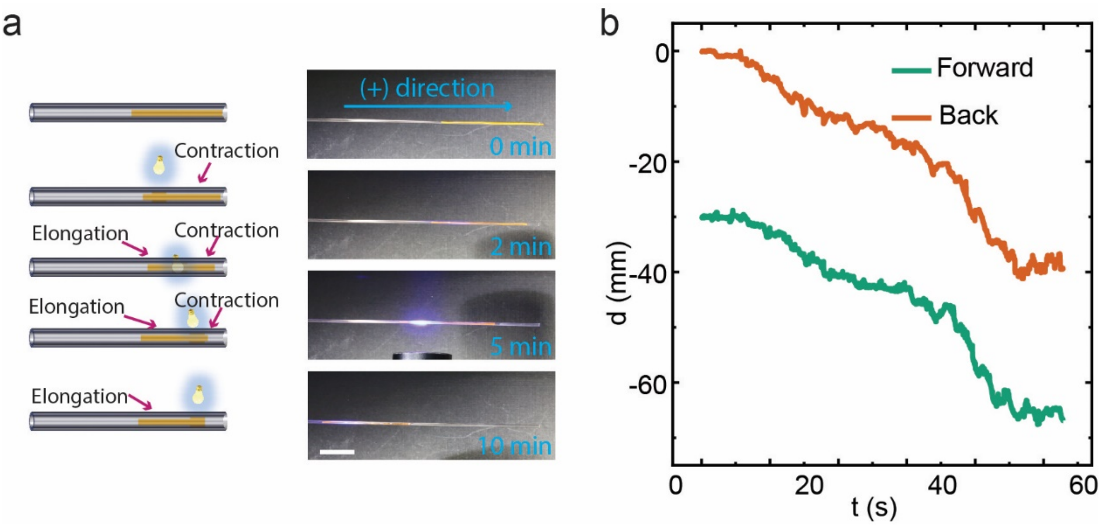

Figure S24. (a) LCE-Azo 15% strip creeping inside a tube under photothermal excitation. (b) Light-fueled creeping distance of front and back sides of the LCE-Azo 15% under modulated light excitation. Irradiation conditions: 460 nm, 1130 mW cm^{-2}. Sample dimensions: 30×0.6×0.5 mm^3. Scale bar: 1 cm.

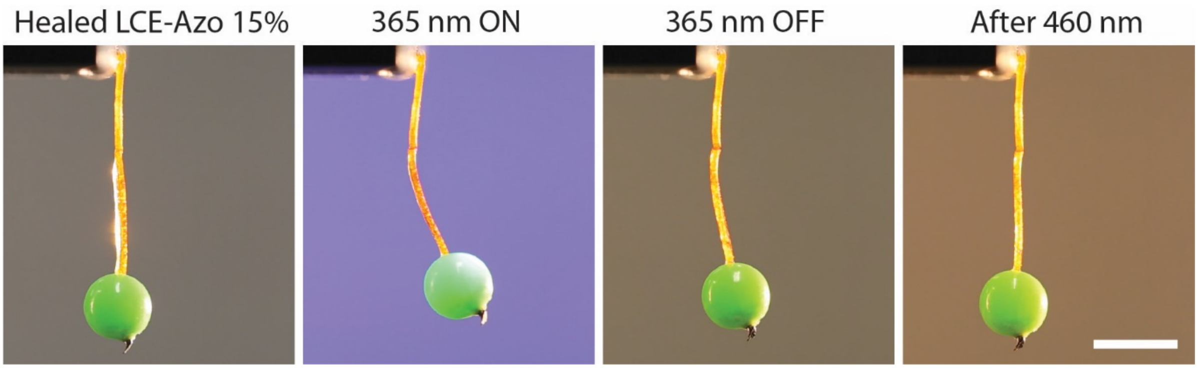

Figure S25. Photochemical actuation of welded LCE-Azo 15% under external load (plastic ball with a mass of 20 mg). Illumination conditions: 365 nm (240 mW cm^{-2}, 1 min) and 460 nm (160 mW cm^{-2}, 1 min). Scale bar: 1 cm.

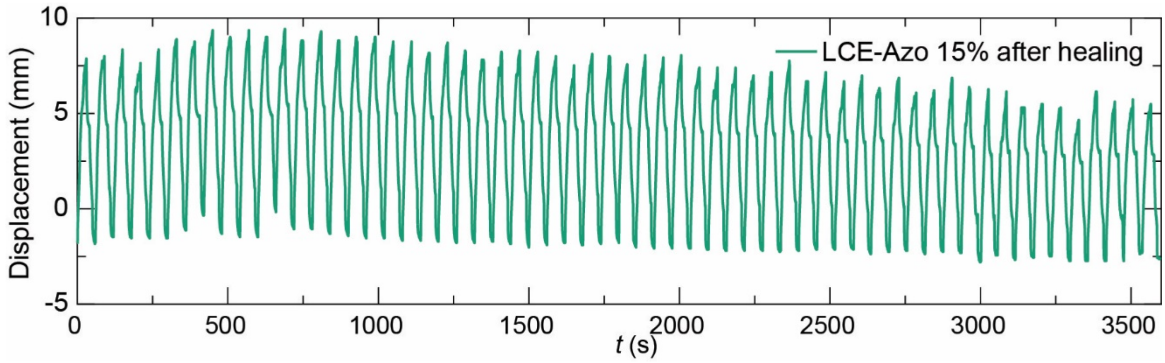

Figure S26. Cyclic photochemical deformation of welded LCE-Azo 15% strip. Illumination conditions: 365 nm, 400 mW cm^{-2}, 460 nm, 160 mW cm^{-2}.

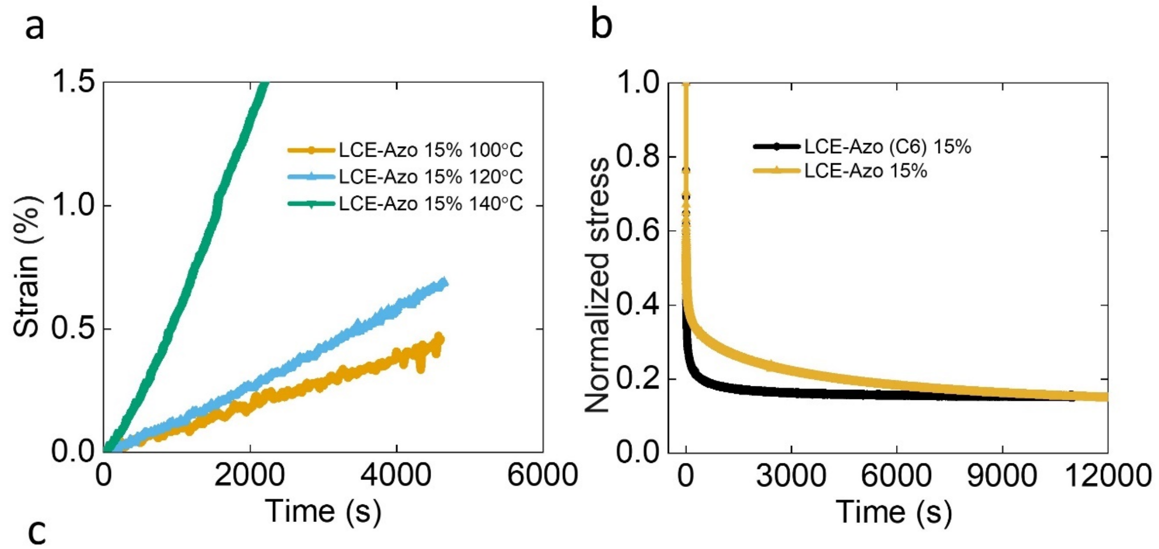

c

	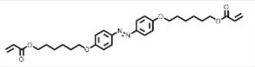	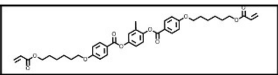	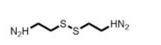	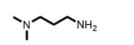	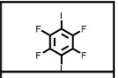
LCE Azo 15%	0.15	0.85	0.3	0.4	0.2
	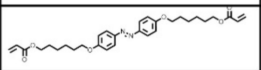	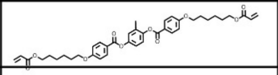	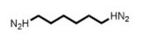	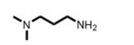	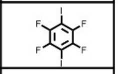
LCE-Azo (C6) 15%	0.15	0.85	0.3	0.4	0.2

Figure S27. (a) Creep curves at different temperature and (b) Stress relaxation curves (measure at 25°C) of LCE-Azo (C6) 15% and LCE-Azo 15%. (c) The chemical composition of LCE-Azo (C6) 15% and LCE-Azo 15%.

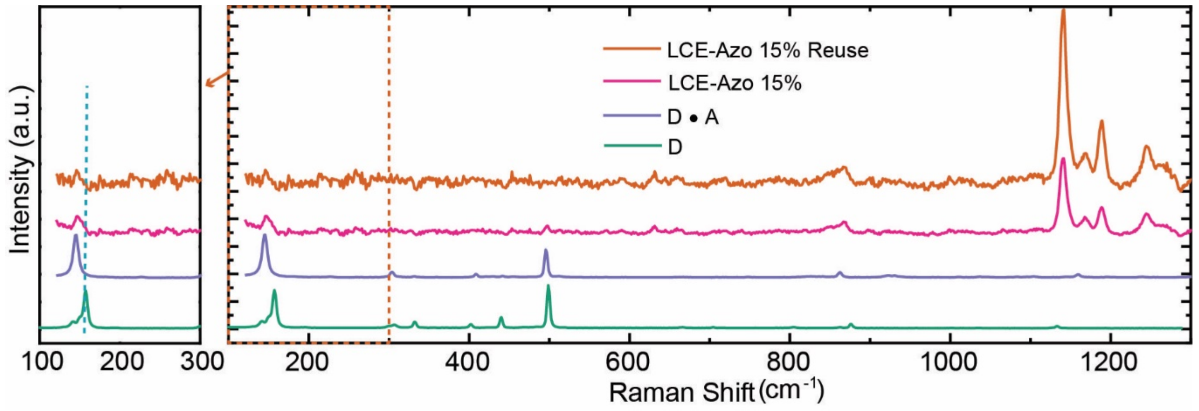

Figure S28. Stacked Raman spectra of **D**, **A·D**, LCE-Azo 15% and LCE-Azo 15% reused after compression molding.

Captions for Movies

Video S1. Photochemical bending (365 nm, 240 mW cm^{-2}) and unbending (460 nm, 160 mW cm^{-2}) of LCE-Azo 15%. Film dimensions: 40×1.0×0.4 mm^3. The movie is played with 4x accelerated speed.

Video S2. The ability of LCE-Azo 15% to lift heavy (10 g or 50 g) objects upon UV (365nm, 320 mW cm^{-2}) or visible (460nm, 1130 mW cm^{-2}) illumination. Film dimensions: 10×1.0×0.4 mm^3. The movie is played with 4x accelerated speed.

Video S3. Photochemical shape programming LCE-Azo 15% to an S shape. Illumination conditions: 365nm, 320 mW cm^{-2}. Film dimensions: 40×1.0×0.4 mm^3. The movie is played with 8x accelerated speed.

Video S4. Light-induced steering of rolling LCE-Azo 15% strip. Illumination conditions: 365 nm, 390 mW cm^{-2}. Sample dimensions: 30×1.0×0.5 mm^3. The movie is played with 8x accelerated speed.

Video S5. An LCE-Azo 15% strip creeping inside a tube. Illumination conditions: 460 nm, 1130 mW cm^{-2}. Sample dimensions: 30×0.6×0.5 mm^3. The movie is played with 4x accelerated speed.

Video S6. Underwater gripper made of LCE-Azo 15%. Illumination conditions: 365nm, 390 mW cm^{-2}; 460 nm, 810 mW cm^{-2}. The movie is played with 8x accelerated speed.

Video S7. Underwater self-rolling of LCE-Azo 15% strip. Illumination conditions: 365nm, 390 mW cm^{-2}. Sample dimensions: 24 × 1 × 0.4 mm^3. The movie is played with 8x accelerated speed.

Video S8. Photochemical actuation of LCE-Azo 15% after welding. Illumination conditions: 365 nm, 240 mW cm^{-2}; 460 nm, 160 mW cm^{-2}. Sample dimensions: 30×1.0×0.4 mm^3.

Video S9. 64 photochemical bending/unbending cycles of welded LCE-Azo 15% strip. Illumination conditions: 365 nm, 400 mW cm^{-2}; 460 nm, 160 mW cm^{-2}. The movie is played with 64x accelerated speed.
